# Mass-Spectrometry-Based Research of Cosmetic Ingredients

**DOI:** 10.3390/molecules29061336

**Published:** 2024-03-17

**Authors:** Alina Florina Serb, Marius Georgescu, Robert Onulov, Cristina Ramona Novaconi, Eugen Sisu, Alexandru Bolocan, Raluca Elena Sandu

**Affiliations:** 1 Biochemistry Discipline, Biochemistry and Pharmacology Department, Victor Babes University of Medicine and Pharmacy Timisoara, Eftimie Murgu Sq. No.2, 300041 Timisoara, Romania; aserb@umft.ro (A.F.S.); sisueugen@umft.ro (E.S.); 2 Physiology Discipline, Functional Sciences Department, Victor Babes University of Medicine and Pharmacy Timisoara, Eftimie Murgu Sq. No.2, 300041 Timisoara, Romania; bolocan.alexandru@umft.ro; 3 Center of Immuno-Physiology and Biotechnologies (CIFBIOTEH), “Victor Babeș” University of Medicine and Pharmacy Timișoara, Eftimie Murgu Sq. No. 2, 300041 Timisoara, Romania; 4 Faculty of Medicine, Victor Babes University of Medicine and Pharmacy Timisoara, Eftimie Murgu Sq. No.2, 300041 Timisoara, Romania; robert.onulov@yahoo.com (R.O.); novaconi.ramona@gmail.com (C.R.N.); 5 Department of Neurology, University of Medicine and Pharmacy of Craiova, St. Petru Rares, No. 2-4, 200433 Craiova, Romania; raluca.sandu@umfcv.ro; 6 Department of Biochemistry, University of Medicine and Pharmacy of Craiova, St. Petru Rares, No. 2-4, 200433 Craiova, Romania

**Keywords:** mass spectrometry (MS), cosmetic ingredients, LC-MS, GC-MS, fragrances, preservatives, dyes, allergens, metals, cosmetics regulation

## Abstract

Cosmetic products are chemical substances or mixtures used on the skin, hair, nails, teeth, and the mucous membranes of the oral cavity, whose use is intended to clean, protect, correct body odor, perfume, keep in good condition, or change appearance. The analysis of cosmetic ingredients is often challenging because of their huge complexity and their adulteration. Among various analytical tools, mass spectrometry (MS) has been largely used for compound detection, ingredient screening, quality control, detection of product authenticity, and health risk evaluation. This work is focused on the MS applications in detecting and quantification of some common cosmetic ingredients, i.e., preservatives, dyes, heavy metals, allergens, and bioconjugates in various matrices (leave-on or rinse-off cosmetic products). As a global view, MS-based analysis of bioconjugates is a narrow field, and LC- and GC/GC×GC-MS are widely used for the investigation of preservatives, dyes, and fragrances, while inductively coupled plasma (ICP)-MS is ideal for comprehensive analysis of heavy metals. Ambient ionization approaches and advanced separation methods (i.e., convergence chromatography (UPC^2^)) coupled to MS have been proven to be an excellent choice for the analysis of scented allergens. At the same time, the current paper explores the challenges of MS-based analysis for cosmetic safety studies.

## 1. Introduction

A cosmetic product is defined by European legislation as a substance or mixture designed to be used in contact with the external parts of the human body. Cosmetic products can be divided into two main categories: leave-on products, which remain in contact with the skin surface after their application, and rinse-off products, which are removed from the skin after a short time period [1,2].

Unlike pharmaceuticals, cosmetics do not play a role in curing diseases. However, modern cosmetics are often “functional” products for wrinkle care, whitening, hydration, and treatment of pores, stains, etc., that are produced to meet consumers’ present needs. Thus, some cosmetics contain quasi-drugs; however, their effects on the body are mild. Some cosmetics manufacturers have begun to use the term “cosmeceutical” to refer to products that have similar benefits to drug use [3]. Still, the new term is not recognized by the FDA (Food and Drug Administration). Instead, the FDA classifies a product as either a drug, a cosmetic, or a combination of the two. Because cosmetics are used freely by consumers who do not have daily exposure limits, the absorption of quasi-drugs (and other ingredients) through the skin must be carefully controlled, making monitoring the transdermal absorption of drugs one of the most essential topics in the field of cosmetic sciences. A wide variety of raw materials that can come from synthetic, biosynthetic, or natural extracts are used in the formulation of personal care products. Suppliers of natural extracts often claim that their products are “organic”, “pesticide-free”, or “natural”, but it is necessary to check whether these statements are, in fact, true. Moreover, these raw materials can be associated with a wide range of contaminants, byproducts, and degradants of the raw materials used in their forms.

Cosmetic formulations include an enormous variety of different types of ingredients, such as active principles, excipients, and additives. Constituents of cosmetics can be generally grouped as ingredients for giving the product form (water, oils, silicones, surfactants, polymers, polyhydric alcohols, inorganic and organic powders), ingredients for stabilizing the products (antimicrobial agents, pH control chemicals, antioxidants, and chelating agents), ingredients for giving efficacies, effects, and concepts (plant extracts and herbal medicine components, microbial-derived ingredients, proteins and amino acids, ceramides, and vitamins) and ingredients acting on the senses of users (coloring and scenting agents) [4]. Ingredients of cosmetics are combined so as to achieve the targeted efficacies and effects and be suitable for the body part on which the product is to be applied, the purpose, and the method of use. For each ingredient, there is a limitation on the quantity that can be included, depending on the body part the product is to be used on and the method of use (to be left on or rinsed off). In addition, there are ingredients whose inclusion is restricted or prohibited due to safety issues [4].

The analysis of cosmetics represents a challenge mainly because of the great variety of ingredients and formulations leading to immense matrix complexity and variability. In this field, many analytical techniques play a crucial role, each designed to meet specific demands to understand the molecular nature of cosmetic products and the complexity of their formulations: chromatography (liquid chromatography (LC) [5,6], gas chromatography (GC) [7,8], supercritical fluid chromatography (SFC) [9], capillary electrophoresis (CE) [10,11], spectroscopy [12,13,14,15,16,17], electrochemistry [18], colorimetry [19,20], mass spectrometry (MS) [21,22,23,24,25,26,27,28,29,30,31], interfacial methods [32,33], rheological assessment [34,35,36], olfactometry, and electronic nose technology [37,38,39]. Recent advances in MS and ionization techniques provide access to richer and deeper information on monitoring the molecular compositions of endogenous or exogenous compounds in or on the skin as well as those used in cosmetic formulations, with less time and effort. In this context, in order to provide safe and efficient products to customers in the cosmetics industry, MS is an indispensable analytical tool. Chromatographic methods hyphenated to MS, such as liquid chromatography–mass spectrometry (LC-MS) and gas chromatography–mass spectrometry (GC-MS), offer the remarkable capability to separate and identify complex mixtures within cosmetic formulations, allowing it to be used for qualitative and quantitative analysis with good sensitivity. However, they often necessitate many instrumental methods for broad coverage of analyte classes and various matrices.

Comprehensive preparation and chromatographic separation can considerably decrease sample throughput, making direct analysis techniques such as ambient MS exciting alternatives to traditional methods [40,41,42,43,44,45,46], mainly when direct assessment of cosmetic efficacy on living organisms (e.g., skin) is required.

In addition to the sensitivity and specificity of the MS method, it is applied to a wide range of compounds, which makes it irreplaceable for the investigation of complex systems required for the development of cosmetics. Analytical measurement for quality control is required to warrant that formulations of cosmetics are in accordance with legislation, with the efficacy and safety of cosmetics being of vital importance and serious concern worldwide.

In the present review, the publications related to the analysis of cosmetic ingredients by MS are presented and examined. Most of the published research articles are based on the hyphenation of chromatography and MS or tandem MS and focus on the assessment of cosmetic ingredients in different formulations, including those restricted or banned. Moreover, the effects of the exposure of human skin to different ingredients are also discussed.

## 2. Literature Research Methodology

The literature search incorporated peer-reviewed journal articles written in English and published between 1992 and 2024, found in the PubMed and Web of Science databases. The main keywords of the search were “mass spectrometry” and “preservatives” and “cosmetic”, “mass spectrometry” and “colorants” and “cosmetic”, “mass spectrometry” and “allergens” and “cosmetic”, “mass spectrometry” and “metals” and “cosmetic”, as well as “mass spectrometry” and “cosmetic ingredients”.

In both databases, the term “All fields” was used, which searches titles, abstracts, author keywords, and keywords plus. Only articles written in English, available full texts, and articles containing publications dedicated to the analysis of cosmetic ingredients by MS are included in this review. Publications containing the analysis of other ingredients than those found in the investigated topic and other analytical methods except MS or MS in conjunction to different separation methods were excluded. Associated data, preprints, meeting abstracts, opinion letters, data papers, notes, editorial materials, and papers not written in English were excluded as well.

Duplicates were removed, and, further, the selection of the publications included in the manuscript was based on the qualitative and quantitative evaluation of articles from the PubMed and Web of Science database, particularly the title of paper, the keywords, the abstract, the year of publication, and then the main text.

## 3. MS Analysis of Cosmetic Ingredients

### 3.1. Analysis of Bioconjugates in Cosmetic Products

The literature data is scarce regarding the investigation by MS of the various bioconjugates existing in cosmetic formulations. A study presented the analysis by matrix-assisted laser desorption/ionization (MALDI) MS of the lipid profile in different cosmetic products (lipsticks and dermatograph pencils), which made it possible to differentiate new products from those in use and those that have expired [47]. The analysis was performed without an elaborate preparation of the samples. For lipstick application, a soft polymer-coated bar was coated with lipstick samples and then “stamped” onto specific stainless steel plates (targets) of the MALDI mass spectrometer. The samples corresponding to the dermatograph pencils were applied directly onto the surface of the plate. The matrix used was α-cyano-4-hydroxycinnamic acid (CHCA) in a solution of concentration 10 mg/mL (50% acetonitrile: methanol) and the samples were covered using a commercial brush [47]. All samples were analyzed in the positive-ion mode. The assignment of chemical structures was performed following the analysis of collision-induced dissociation (CID) MS/MS fragmentation spectra and calculations with Mass Frontier software (v. 6.0, Thermo Scientific, Carlsbad, CA, USA). Analysis of the data obtained showed that lipsticks often have a formula rich in a very complex lipid matrix (up to 90%), such as Ricinus communis oil (castor oil), beeswax, Candelilla wax, Carnauba wax, lanolin, etc.

These lipid matrices are composed of a wide range of lipid classes, generally triacylglycerols (TAG), sphingolipids (SP), free fatty acids, and fatty esters, among many others. This great variability in composition is what gives these products particular characteristics such as fineness, thickness, creamy texture, and even the thixotropic effect. When analyzing and comparing different types of lipsticks (new, in use, and expired), a different composition of lipids was observed, especially in terms of oxidation of TAGs and the presence or absence of lipids with higher complexity, such as ceramides: the presence of cleaved species in expired lipsticks, such as diacylglycerols (DAG) which are usually in the range *m*/*z* 500–600, for “in use” lipsticks showed several types of TAGs, while “new” ones contained sphingolipids (ceramides) as the main difference from the others [47]. Similar observations were noted for dermatograph pencils: the only notable difference observed was the presence of oxidized TAGs in expired products and, due to the introduction of oxygen species into molecules (epoxy, keto, and hydroxy acids), the *m*/*z* range increases (~900 and higher). The ions from *m*/*z* 700–800 demonstrate the presence of TAGs in both new and “in use” samples. The difference between “in use” and expired samples of dermatograph pencils and lipsticks in terms of their compositions may be due to the fact that lipsticks are usually in contact with saliva and other compounds around the mouth area; therefore, other chemical transformations are more likely to occur than oxidation, compared to dermatograph pencils [47].

In order to determine the commercial ceramides (from natural extracts, Doosan Corporation Bio, Seoul, Republic of South Korea) in cosmetics for the quality control of the product formulation, a fast, sensitive and selective method was used that involves the coupling of reverse-phase liquid chromatography (LC) with electrospray ionization (ESI)-MS [48]. Using LC/ESI-MS with fragmentation at source by CID in both positive- and negative-ionization modes, it was possible to separate and identify the structures of sphingoid bases (phytosphingosine at *m*/*z* 267, 255 and 225 and sphingosine at *m*/*z* 263 and 237) and the N-acyl chains of ceramides, as well as an impurity. The VG MassLynx MS software (https://www.waters.com/nextgen/us/en/products/informatics-and-software/mass-spectrometry-software/masslynx-mass-spectrometry-software.html) used could switch between positive- and negative-ion modes within the same HPLC run, and at higher cone voltages, in both modes, they yielded fingerprint spectra providing complementary structural information about both the fatty acid and the sphingoid base moiety.

It was observed that ceramides were separated and detected with a higher degree of sensitivity in the positive-ion mode than in the negative-ion mode, while the product ion spectra in negative-ion mode of ceramide species provided more structural information than those obtained in positive-ion mode [48]. This study is one of the few that investigates ceramides present in cosmetics.

Ceramides are involved in the skin’s barrier function, with their low levels in the intercellular lipid lamellae of the stratum corneum being associated with dry skin [49]. In practice, certain ceramides applied individually or as an emulsion mixture synergistically improve the skin barrier function in humans [50]. Moreover, some dominant emulsions with ceramide or pseudoceramide can decrease the severity of pruritus and trans-epidermal water loss in various subjects [51]. Recently, ceramides have also been reported as one of the main constituents of topical formulations for rosacea [52] and atopic facial eczema [53]. Although the use of ceramides in cosmetics is widespread, more extensive studies on the toxicity and effects of topical administration of cosmetics containing these types of lipids are needed. MS-based methods are indispensable tools for cosmetic science, since the molecular composition on the surface and in most of the skin is extremely complex and difficult to elucidate.

In recent years, research in the field of biosurfactants has begun to intensify due to the great potential for their use in various branches of the economy, industry, and medicine. Biosurfactants can be used as emulsifiers, de-emulsifiers, softeners, dispersants, foaming agents, active food ingredients, and detergents in various industrial sectors such as oil, organic chemistry, food, cosmetics and pharmaceuticals, mining and metallurgy, fertilizers, environmental protection, etc. In this sense, some glycolipids have particular properties such as surfactant, gelling, and antimicrobial, and as a result, these glycolipids are increasingly used not only in pharmaceutical applications but also in the cosmetics industry [54,55]. Due to all the properties listed above, as well as biodegradability and biocompatibility, sucrose ester-type glycolipids are used in many cosmetic applications.

These glycolipids include the following:

- Lipids with manosyl erythritol (MEL)—these are glycolipids composed of a fatty acid ester, either 4-OD-manopyranosyl-erythritol or 1-OD-manopyranosyl-erythritol [56], produced by yeasts of the genus Pseudozyma, which have been shown to have a moisturizing action compared to natural ceramides on the skin. These glycolipids are used in antiwrinkle and skin-smoothing cosmetics [57].
- Sophorolipids (SLP)—these are glycolipids composed of fatty acids of 16 or 18 carbon atoms bound to a sophorose as a hydrophilic part, produced by several species of Candida or other related yeast species. These glycolipids are used in detergents, lipsticks, lip creams, and eyeshadow [58].
- Trehalose lipids—these are glycolipids composed of fatty acids linked to a disaccharide, trehalose, which is a nonreducing disaccharide in which two glucose molecules are linked in an α, α, 1,1-glycosidic bond.

With regard to polysaccharides, their use in cosmetics has been as pervasive as the use of cosmetics. Historically, due to their rapid availability from common natural sources and their varied and unique multifunctionality, polysaccharides have been included in cosmetics for centuries; for example, the use of β-glucan derived from yeast extracts is used as a natural healing agent. Today, polysaccharides play an even more significant role in the technology of formulating cosmetics. The interaction of polysaccharides with other ingredients in a formulation (e.g., actives, surfactants, salts, other polymers, etc.) and the ease with which they can be chemically modified allowed their pre-eminent use in cosmetics. In addition, polysaccharides, of natural origin and polymeric, are renewable and do not have a safety profile, since that is not accorded to synthetic polymers. When considering the many cosmetically acceptable polysaccharides, it is found that their morphology and functionality cover the entire territory of polymer technology. Polysaccharides perform a multitude of cosmetic functions. For example, they act as rheology modifiers, suspending agents, hair conditioning agents, and wound healing agents. They moisturize, hydrate, emulsify, and hemolyze. Trying to distinguish the influence of a single polysaccharide in a formula is like trying to understand the action of a finger while ignoring the hand as a whole.

Dextrins are a class of low-molecular-weight carbohydrates produced by the acidic and/or enzymatic partial hydrolysis of starch or glycogen, with the structure α-(1 → 4)-Glucose (Glc) of amylose and the branched structure α-(1 → 4)-Glc and α-(1 → 6)-Glc of amylopectin, but with lower polymerization. Converting starch to dextrins is a simple and inexpensive method of reducing thickening and accelerating its moisturizing properties. Dextrin-containing solutions are clear but have a much lower viscosity than parental starch. Dextrins have a variety of uses as absorbents, binders, fillers, adhesives, films, conditioning agents, thickeners, or as a foundation for makeup and face powders. Dextrins are also used as an aid for spray drying or encapsulation to provide new dosage forms, controlled-release or tasteless drugs, and food flavorings. Dextrins are an accessible raw material, generally considered safe [59]. Regarding dermal cosmetics and biomedical applications, the use of dextrins is still relatively unexplored; they are used clinically as peritoneal dialysis solutions that can also act as drug delivery solutions [60] and as a wound dressing agent. Dextrins have a set of advantages that enhance their specific use in biomaterials: they are biocompatible and nonimmunogenic materials, degradable in vivo by amylases, and their molecular weight ensures renal elimination, thus avoiding their accumulation in tissues due to repeated administration [61].

Regarding the analysis of carbohydrate polymers, MALDI-TOF MS has proven to be an accurate technique for characterizing their molecular weight distribution [62,63]. Kazmaier et al. [64], studied a set of maltodextrins with a degree of polymerization in the range of 2 to 13 and concluded that MALDI-TOF MS is the most suitable technique for detecting high-molecular-weight species such as oligosaccharides. Thus, it was observed that the degree of polymerization increases linearly with the molecular weight [64,65]. Silva et al. [66] reported comprehensive structural characterization of several commercial dextrins, which were used to produce adipose dihydrazide crosslinked oxidized dextrin hydrogels by MALDI-TOF in the positive-ion reflector mode using delayed extraction in the mass range between 600 and 4500 Da, and size exclusion chromatography (SEC). MS characterization provided important data on the chemical structure of different maltodextrins, determining the number of glucose oligomers (6–17) contained in carbohydrate polymer chains (degree of polymerization, DP), which is essential to establish potential applications for commercial maltodextrins [65].

### 3.2. Analysis of Preservatives in Cosmetic Products

**Figure 1:**
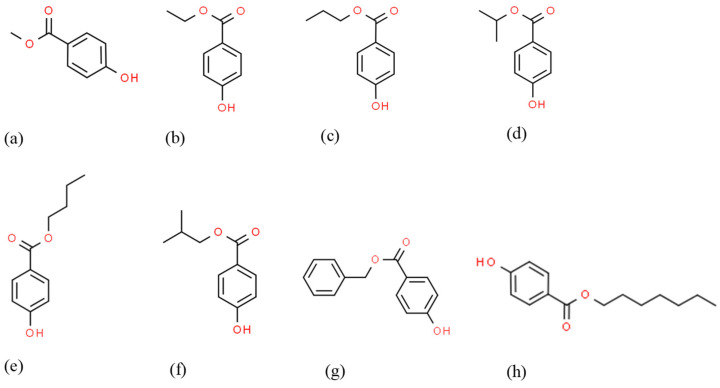
Chemical structures of parabens found in cosmetic products. (**a**) MP; (**b**) EP; (**c**) PP; (**d**) IPP; (**e**) BP; (**f**) IBP; (**g**) BeP; (**h**) HP.

Parabens are esters of the parahydroxybenzoic acid (methylparaben (MP), ethylparaben (EP), propylparaben (PP), butylparaben (BP), isobutylparaben (IBP), isopropylparaben (IPP), benzylparaben (BeP), and heptylparaben (HP) (Figure 1)), which, due to their low volatility, high stability, and their antibacterial and antifungal properties, have been the most used as preservatives in cosmetics, personal care, pharmaceuticals, food, and industrial products.

Parabens have traditionally been considered low-toxicity compounds. However, it has been discovered that some parabens can function as endocrine disruptors, leading to a potential increase in the incidence of breast cancer in women or the onset of malignant melanoma. They have also been associated with contact dermatitis and rosacea. These risks are further exacerbated by the ability of parabens to be absorbed by human skin without being degraded by esterases [67]. Since they are unregulated, they can contain levels of preservatives that pose a health risk, which is doubly true for counterfeit cosmetics. Therefore, accurate methods must be used to determine the levels of these compounds in cosmetics and personal care products. Current analytical methods based on MS for the determination of preservatives in cosmetics and personal care products include high-performance liquid chromatography (HPLC) and ultra-high-performance (UHPLC) coupled with mass spectrometry (HPLC-MS and UHPLC-MS), as well as gas chromatography coupled with mass spectrometry (GC-MS). For analyte quantitation, internal standards (ISs) can be used. These are chemical substances which are added at the same concentration to all samples throughout a quantitative analysis. The main criteria for choosing an internal standard is based on resolution—the IS should not be present within the sample matrix or interfere with any other compounds present within the sample. Ideally, a compound which is similar in nature to the target analyte(s) would be chosen, as this is likely to behave in a very similar way, giving a similar retention time, peak shape, and response. It is very common in the GC-MS method for the deuterated form of the target analyte to be used. For complex analysis with a large number of components, multiple ISs can be used to calculate analyte concentrations throughout the method. Using an IS is a powerful tool for minimizing the effects of random and systematic errors during analysis, helping to improve the precision of results and reduce the need for repeat measurements.

#### 3.2.1. HPLC-MS and UHPLC-MS

HPLC-ESI-MS using ionization in both positive- and negative-ion mode and scanning in the mass range of *m*/*z* 100–1000 was applied to determine MP, EP, PP, and BP, butylated hydroxyanisole (BHA), and butylated hydroxytoluene (BHT), as well as α-tocopherol (α-t) and α-tocopherol acetate (α-ta) in various cosmetics (a commercial lanoline cream, a commercial skin milk, and a commercial cream) after supercritical fluid extraction procedure (SFE) and separation of the analytes on a C18 reversed-phase column using methanol–water as mobile phase [68]. Pseudomolecular ions [M − H]^−^ or [M + H]^+^ were obtained as the base ions. MP, EP, PP, BP, BHA, and BHT were easily deprotonated at the electrospray ion source under the experimental conditions to form the negative molecular ions [M − H]^−^, and, therefore, ESI in the negative-ion mode was selected as the best ionization mode and employed for four paraben preservatives, BHA and BHT. ESI in the positive-ion mode was employed for α-tocopherol and α-tocopherol acetate. The base ions were used to quantify each compound to increase sensitivity in the selected ion monitoring (SIM) mode. This method is accurate and reliable for all analytes and made it possible to determine their concentrations in real commercial cosmetics from not detected to 977 mg/kg.

Nevertheless, it is challenging to obtain data on the use of cosmetics and the biological (e.g., human blood serum) concentrations of their active ingredients. Using LC-ESI-MS/MS, it was possible to determine MP and PP concentrations in serum and correlate the results with the routine application of oral cosmetics (lipstick) [69]. Tahan et al. [69] analyzed blood samples (15 mL) of volunteer women for three phases: the women used paraben-containing products according to their routine (phase 1), the women used lipsticks containing MP and PP for five days in conjunction with the routine use of paraben-containing products (phase 2), and in phase 3, the women routinely used paraben-containing products while refraining from using lipstick for five days. A statistically significant difference between serum parabens concentration in women who used lipstick containing these preservatives compared to the absence of this cosmetic in their daily routine was demonstrated, and a strong association between serum parabens and lipstick use was observed.

While the methods used for the preservative analysis of cosmetic products have mainly focused on the determination of parabens, the analysis of more than one class of preservatives is still a field under development. Using LC-ESI-MS in addition to MP, EP, PP, and BP, four other preservatives, BeP, butylated hydroxyanisole (BHA), DL-α-tocopherol acetate, and 2,6-di-tert-butyl-4-methylphenol (BHT), could be separated, identified, and quantified in samples of 100 mg of cosmetic and personal care products (lipstick, foundation, deodorant, hand lotion, soap, and toothpaste). BHT, MP, and EP were detected in most samples, while BeP and BP were not detected in any of the tested samples. It is interesting to note that the deodorant sample tested and one of the foundations had relatively higher amounts of parabens (MP and EP) compared to the other products. PP was also detected in the foundation sample. It was observed that some of the analyzed products also contained peaks belonging to preservatives that were not listed on the list of ingredients on the product label. In comparison with other literary references, this method combines a simplified and inexpensive sample preparation procedure with a short analysis time (8 min) while providing a similar, if not improved, degree of separation and sensitivity [70].

Methods based on UHPLC-ESI-MS have been used more to determine parabens (MP, EP, PP, and BeP) as contaminants in ambient waters in only 9 min of chromatographic separation [71].

Moreover, for monitoring long-term exposure to parabens, an improved analytical method based on UHPLC-ESI-MS for rapid and direct determination of parabens in hair samples was successfully developed and implemented using online extraction and negative ionization. Hair samples (*n* = 10) were cut into pieces of 1–2 mm and placed in methanol/dichloromethane (1:1) solution, extracted by ultrasonication at room temperature for 30 min twice, and subjected to centrifugation, after which the supernatant was injected into the LC-MS/MS. Five parabens (MP, EP, PP, BP, and BeP) were, thus, detected and quantified (Table 1) in hair samples by LC-MS/MS using online extraction and were completely separated in MRM mode. All of the parabens were observed as the deprotonated precursor ions of [M − H]^−^ type, and for the generation of MS/MS spectra, deprotonated precursor ions were fragmented at optimized parameters and the product ions were generated from the loss of either the alkyl chain or benzyl groups from the ester group (*m*/*z* 136 ion), followed by the loss of CO_2_ (*m*/*z* 92 ion)—the most abundant product ion of MS/MS for all the parabens which was chosen for quantification, whereas *m*/*z* 136 ion (the second highest ion) was used as confirmation for the parabens [72].

All of the parabens were observed as deprotonated precursor ions [M − H]^−^, which were subjected to fragmentation by MS/MS. The obtained product ions resulted from the loss of either the alkyl chain or benzyl groups from the ester group (*m*/*z* 136 ion—the second highest ion which was used as confirmation for the parabens), followed by the loss of CO_2_ (*m*/*z* 92 ion—the most abundant product ion of fragmentation for all the parabens, which was chosen for quantification).

The developed method has been shown to be sensitive, selective, and accurate and was appropriate in hair sample analysis [72].

**Figure 2:**
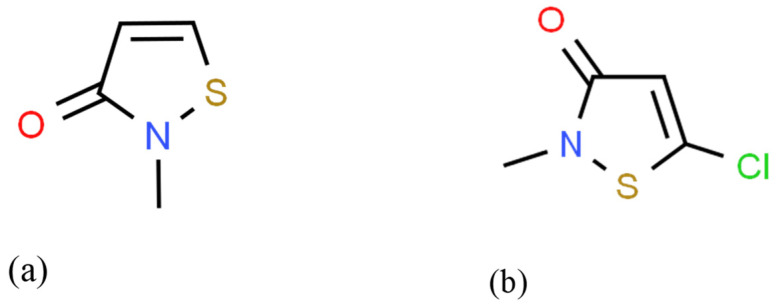
Chemical structures of (**a**) MIT (C_4_H_5_NOS) and (**b**) MCI (C_4_H_4_ClNOS).

Other compounds used as common preservatives in many personal care products (mascara, makeup remover, liquid soaps, body wash, hairspray, hair color, conditioner, shampoo, lotion, baby shampoo, baby lotion, sunscreen, shaving cream, and detergents) are methylisothiazolinone (MIT) and methylchloroisothiazolinone (MCI) (Figure 2).

MIT and MCI inhibit bacterial growth in cosmetic products on their own, but are most commonly used as a mixture in products. Their presence in different cosmetic products has been linked to allergic reactions, lung toxicity, and possible neurotoxicity [73] and has been investigated over time using GC-MS, LC-MS, and HPLC-UV. In recent years, an efficient UHPLC-MS/MS method was developed and validated for the determination of MIT and MCI in selected cosmetic products (shampoo/conditioners or skin care products, such as body lotions, gels, moisturizers, and body cleansers) by Wittenberg et al. [74]. The method used in this study optimizes the extraction and chromatography parameters of Lin et al. [75] by using four columns (Waters Acquity BEH C18, Waters Acquity BEH Amide, Agilent Poroshell 120 PFP, and Phenomenex Kinetex HILIC) in addition to the column used by Lin et al. (Waters Acquity HSS T3) to increase the retention times of the analytes and achieve an efficient determination of only MIT and MCI in cosmetic products. However, the Acquity HSS T3 column in combination with H_2_O w/0.1% formic acid (FA) and MeOH w/0.1% FA as the mobile phases was selected for separation because it produced optimal peak shape, sensitivity, and retention times. Thus, the mass spectrometer was operated in scheduled multiple reaction monitoring (MRM) mode and used positive ESI as the ionization source. A stock solution containing 5 g/mL of MIT and MCI in 50:50 H_2_O/acetonitrile *v*/*v* and stock IS solution containing 250 ng/mL of MCI-d3 in 50:50 H_2_O/acetonitrile *v*/*v* were prepared separately in different volumetric flasks. Ten calibration solutions were prepared using the stock solution. The concentration ranges for the standard solutions were 0.1–500 ng/mL for MIT and 0.1–1000 ng/mL for MCI. A constant concentration of 25 ng/mL of the IS was added to each standard solution and was used for quantitation by plotting the ratio of analyte signal to internal standard signal against the concentration of the analyte. Analyte confirmation was determined by the primary transition to secondary transition ratio. Any value within 15% of the theoretical ratio (0.734) was confirmed as a true-positive result.

The lower limit of quantitation was determined to be 0.1 g/g for both preservatives. The concentrations of MIT and MCI ranged from not quantified, or below the lower limit of quantitation, to 89.64 g/g and not quantified to 10.31 g/g, respectively.

The analytical method described by Wittenberg et al. [74] was proved to be the fastest and most sensitive method for identifying and quantifying MIT and MCI in cosmetic products and may be applied to a wide variety of cosmetic products, being suitable for monitoring the frequency of incorrect labeling of MIT and MCI on cosmetic product ingredient lists.

#### 3.2.2. GC-MS

In recent years, GC-MS has been increasingly applied to paraben analysis, competing with traditional HPLC-UV in terms of the number of publications and even surpassing it in terms of application to environmental analysis. The proposed GC-MS methods for the determination of parabens are based on a variety of mass analyzers: Q [76,77,78,79,80], triple Q [81], IT [82], and TOF [83]. In general, GC-MS has the same advantages as HPLC-MS: unambiguous identification of analytes and low detection limits that allow the determination of parabens present in low concentrations and their simultaneous determination with other species of various natures. GC-MS also has some advantages over HPLC-MS, with higher resolution, lower costs, and lower solvent waste production. On the other hand, GC-MS usually requires derivatization of the analytes to obtain their corresponding volatile derivatives. Thus, the GC-MS methods for the determination of parabens can be divided into three groups: (a) based on derivatization by acetylation with acetanide—applied to the determination of parabens and other preservatives in soaps, shampoos, makeup products, creams, body milk, etc. [82]; (b) based on derivatization by silylation with N, O-bis (trimethylsilyl) acetamide—applied for the determination of parabens in water and cosmetics [76]; (c) without derivatization, in the form of GC-MS [84] and GC-MS/MS [81]. In the latter case, isotopically labeled versions of these parabens were used as IS for quantitation to compensate for the fluctuation in instrument response and matrix effects in complex matrices such as cosmetic products. A stock standard solution of parabens was prepared in MeOH to a concentration of 5000 g/mL. Working standard 1 was prepared by transferring 1 mL of the MP stock solution, 2 mL of the EP stock solution, 4 mL of the PP stock solution, and 5 mL of the BP stock solution into a 20 mL scintillation vial. Working standards 2 and 3 were prepared by diluting working standard 1 8-fold and 50-fold with MeOH, respectively. A stock IS solution was prepared by weighing each paraben-d4 standard into a volumetric flask, to which MeOH was added up to a concentration of 4000 g/mL. A working IS was prepared by transferring 1, 2, 4, and 5 mL of MP-, EP-, PP-, and BP-d4 stock standard solution into a vial and mixing well. Both working standards 2 and 3 and the working IS were used to prepare calibration standards in MeOH. Eight point calibration curves using peak area ratios were created. The concentrations ranged from 50.0 to 10,000 ng/mL, 100.0 to 20,000 ng/mL, 200.0 to 40,000 ng/mL, and 250.0 to 50,000 ng/mL for MP, EP, PP, and BP, respectively. IS concentrations in each calibration standard were 1000, 2000, 4000, and 5000 ng/mL for of MP-, EP-, PP-, and BP-d4, respectively.

The measurements were performed using dynamic monitoring of the selected reaction (automatic selection of the optimal scanning parameters for each analyte during elution, via software); this procedure led to an improvement in the peaks corresponding to the MS analysis of the samples, further reducing the loss of resolution caused by the absence of a derivatization reaction.

### 3.3. Analysis of Colorants in Cosmetic Products

Due to the hydrophilic nature of most dyes, LC is the usual choice in combination with MS for their investigation. Chromatographic separation is often required to properly identify and quantify dyes in cosmetic samples. Due to the ability of dyes to absorb in the UV–Vis spectrum, diode-array (DAD) or UV–Vis detectors have traditionally been the preferred detectors. In recent years, MS has become a valuable choice [23,85,86,87,88], given the increased selectivity and sensitivity that are especially useful in the analysis of banned compounds. MS exceeds DAD limitations, such as the overlap of UV–Vis spectra between matrix ingredients.

Largely, acid (possessing acidic groups, such as SO_3_H and COOH and of anthraquinone-, azo-, and triarylmethane type), direct, and fiber-reactive dyes are manufactured as salt compounds, are typically anionic, and ionize in negative-ion mode in MS, while basic dyes (usually salts generated by aromatic bases reacting with acids) are cationically charged and ionize in positive-ion mode in MS; disperse dyes (mostly polar molecules containing azo or anthraquinone groups) generate positive ions during mass spectrometric analysis, but the dye itself is nonionic [89].

In some studies, a comparison between LC-DAD and LC-MS/MS [85,88] was established. In this way, Noguerol et al. [88] developed two LC methods: with UV–Vis detection and MS in tandem with triple quadruple with ESI ionization performed in positive-ion mode for routine control of 10 dyes (Fat Brown RR, Malaquite Green Carbinol Base, Dimethyl Yellow, Sudan I, Sudan Orange G, Solvent Blue 35, Sudan II, Sudan Black, Sudan III and Sudan IV), especially dyes banned in the cosmetics industry. LC-ESI-MS/MS in full mass spectrum scanning mode allowed structural information to be obtained about multiple peaks observed for some of the dyes studied by HPLC-UV/Vis analysis. The presence of more than one peak for a compound was mainly due to possible isomerization processes, impurities, or degradation products. Multiple reaction monitoring (MRM) mode was used for quantification. In terms of sensitivity, there was a remarkable difference between the two methods. The quantification limits were one or two orders of magnitude smaller for the LC-MS/MS analysis, which is particularly appreciated given the degree to which these dyes are restricted in commercial products. The combination of LC and MS in the analysis of cosmetic dyes was first reported by Xian et al. [87] in 2013. Subsequently, Guerra et al. have developed several improved methods based on this analytical technique [23,85,86].

Using sequential steps based on HPLC, LC-MS/MS, and LC-Q-TOF-MS, in Han et al. [90], 13 banned synthetic colorants were detected and structurally characterized (Basic Blue 26, Basic Red 2, Disperse Brown 1, Disperse Orange 3, Disperse Yellow 3, HC Blue No. 2, HC Yellow No. 5, Solvent Orange 4, Solvent Yellow 1, Solvent Yellow 3, Solvent Orange 7, Solvent Red 24, and Basic Yellow 28) in 120 purchased long-lasting cosmetic samples (classified as tattoo eyebrow, tattoo lipstick, and hair tint). First, sample solutions were prepared with 100% methanol to a volume of 50 mL and subjected to ultrasonic extraction at room temperature for 30 min, followed by centrifugation and filtration. As the next step, the illegal colorants were detected by HPLC with diode-array detection (HPLC-DAD) using an Agilent 1260 Infinity II LC system (Agilent, Santa Clara, CA, USA) equipped with a DAD and separated on a Zorbax Eclipse XDB-C18 (4.6 mm × 150 mm, 5 μm; Agilent, Santa Clara, CA, USA) column and by LC-MS/MS performed on a Waters ACQUITY ultra-performance liquid chromatography coupled with a Xevo TQ-XS (Waters, Milford, MA, USA) system using ESI as ionization in both positive- and negative-ion modes. The mobile phase consisted of 5 mM/10 mM ammonium acetate in water containing 0.1% formic acid and acetonitrile–MeOH (80:20, *v*/*v*). Disperse Yellow 3 and HC Yellow No. 5 were detected in the negative-ion mode, while the other 11 compounds were detected in the positive-ion mode.

The MS fragmentation patterns, confirmed via LC-Q-TOF-MS (performed using an Agilent 1290 Infinity II LC system (Agilent Technologies, Santa Clara, CA, USA) coupled with an Agilent 6545XT Q-TOF-MS system (Agilent Technologies, Santa Clara, CA, USA)), of 11 out of 13 prohibited dye species were reported by Han et al. [90] for the first time, and among the 120 cosmetic samples, one was found to contain three illegal compounds: Basic Blue 26, Basic Red 2, and Basic Yellow 28. Thus, the work of Han et al. [90] shows future promise in view of more rigorous screening and control of the presence of illegal ingredients in cosmetic products and to hinder/restrain their distribution.

Most dyes used in cosmetics are sodium or calcium salts which contain one or more ionized groups in their structure, such as sulfonic groups. This implies the possible formation of multicharged ions in the ionization source. In addition, the separation of ionic compounds by reverse-phase LC is a difficult task that requires significant effort, especially in the separation of neutral compounds. In this sense, the mobile phase (ionic strength, pH, and composition) plays an important role. In some cases, a mobile phase without additives, consisting of water and an organic modifier (acetonitrile or methanol), was used with good results—good sensitivity and rapid analysis [86,87]. The use of UPLC-MS/MS allowed the analysis of 11 dyes (including Acid Violet 49, Pigment Red 57, Pigment Red 53:1, Acid Yellow 36, Rhodamine B, Basic Violet 3, Disperse Yellow 3, Pigment Orange 5, Sudan Ⅰ, Sudan Ⅱ, Sudan Ⅳ, and Solvent Blue 35) in 4 min [18] and, respectively, of 12 dyes (including Tartrazine, Amaranth, Ponceau 4RC, Sunset Yellow, Allura Red AC, Acid Red 2G, Ponceau SX, Brilliant Blue FCF, Orange I, Acid Black 1, and Acid Orange 7) in 6 min [91] in lip gloss, eyeshadow, lipstick, and other cosmetics. The results showed that the UPLC-MS/MS method could be a fast, simple, sensitive, and quantitative technique for the simultaneous determination and confirmation of dyes in oily cosmetics, cream cosmetics, and powder cosmetics. Similar results were obtained for a mixture of nine dyes with a conventional porous C18 column [86].

Although the use of MS allows selective identification of coeluted compounds, chromatographic separation is recommended. To this end, it is necessary to add volatile neutral salts to the mobile phase to avoid interactions between negatively charged ionized compounds and partially ionized residual silanols in the stationary phase. However, the presence of salts in the ion source may cause a suppression of ionization. Thus, the composition of the mobile phase must be investigated to achieve a compromise between good separation and performance. Therefore, the use of only 3 mM ammonium acetate in the mobile aqueous phase is recommended [23,85]. This salt concentration was sufficient to avoid “peak tailing”, while the improvement of chromatographic separation for a fairly large number of analytes was within the satisfactory quantification limits. In another study [85], other chromatographic parameters were optimized to separate dyes from preservatives. The matrix effect is the suppression or amplification of the ionization of the target compound by others in the sample and is very common in LC-MS/MS analysis, especially when ESI sources are used. In each method of dye analysis by MS/MS, a matrix effect study was performed. The most comprehensive study was performed for 19 dyes in 7 cosmetic matrices (lip balm, nail polish, hair spray, eyeshadow, toothpaste, blush, and gel) [23]. In all cases, the optimized sample extraction procedure allowed a sufficiently clean extract to perform the analysis with negligible matrix effects, except for certain compounds in several matrices. Unlike conventional techniques of MS, Nizza et al. [92] investigated the use of MS-coupled desorption electrospray ionization (DESI) for the analysis of semipermanent hair dyes in two semisolid cosmetics: a blemish cream (BB cream) and a hair coloring gel. As a novelty, the use of an environmental MS technique allowed a direct analysis without prior sample preparation or chromatographic separation. A thin layer of sample is deposited on the porous Teflon, and a pneumatically assisted ESI is used to release neutral analytes present on this surface as secondary ions. To test the robustness of direct DESI-MS analysis towards complex chemical matrices, a 10-component mixture was deposited onto a surface and examined in both positive- and negative-ion modes. Thus, positive-mode DESI mass spectrum resulting from this analysis yielded protonated molecules for o-toluidine (*m*/*z* 108), p-phenylenediamine (*m*/*z* 109), resorcinol (*m*/*z* 111), 2,4-toluenediamine (*m*/*z* 123), 4-chloroaniline (*m*/*z* 128), benzidine (*m*/*z* 185), and benzyl salicylate (*m*/*z* 229), while the azo dyes Ponceau SX (PSX), Sunset Yellow FCF (SY FCF), and Orange II (OII) were readily detected in negative-ion mode, at *m*/*z* 217 [M − 2Na]^2−^, 435 [M + H − 2Na]^−^, and 457 [M − Na]^−^ for PSX, *m*/*z* 203 [M − 2Na]^2−^, 407 [M + H − 2Na]^−^, and 429 [M − Na]^−^ for SY FCF, as these sulfonated azo dyes are disodium salts and only the nonsodiated anion at *m*/*z* 327 for OII.

MALDI ionization coupled to MS has proven to be a fast and robust technique for the evaluation and quantification of compounds of interest in various common cosmetic matrices. In this sense, this has been taken as far as creating a new subdomain—“Cosmetomics”, as a simple alternative, both for industrial and academic analysis, using the MALDI-MS principles for the purpose of product analysis. By using MALDI-MS, in the work of Oliveira et al. [47], the quantification in nail polishes of the dye Sudan III (with potential carcinogenic risk), which is a common dye in cosmetics, was performed. It was declared as an ingredient on all labels as CI 21600 (color index) and/or Solvent Red 23 (trade name). The health risks associated with a possible carcinogen in a nail polish formulation are due to accidental ingestion through nail biting or even during cooking or baking.

The analysis was performed without complex preparation of the samples, these being applied directly on the surface of the MALDI board. The matrix used was α-cyano-4-hydroxycinnamic acid (CHCA) (Sigma Aldrich, Allentown, PA, USA) in 10 mg/mL solution (50% antonitrile: methanol) and the samples were coated using a commercial brush [47]. MALDI-MS analysis was performed using an MALDI-LTQ-XL with imaging feature (MSI) (Thermo Fisher, Pleasanton, CA, USA) and MS/MS experiments were performed by collision-induced dissociation (CID) in negative-ion mode. The assignment of chemical structures was conducted following the analysis of MS/MS spectra, as well as by calculations with Mass Frontier software (see 6.0, Thermo Scientific, Carlsbad, CA, USA). Image data were analyzed in triplicate using ImageQuest software (Thermo Scientific, Carlsbad, CA, USA, https://www.thermofisher.com/order/catalog/product/10137985) and quantification was performed using ImageJ (National Institutes of Health, USA-Open Source) on grayscale images. The area was standardized in the number of pixels for all reproductions, and ImageJ software (https://imagej.net/ij/) assigned a value for selection based on the intensity of each pixel [47]. The results obtained proved that this approach is a useful, fast, and easy tool for semiquantitative analysis. The results obtained were promising, and consistent because the amount of Sudan III (*m*/*z* 351, [M − H]^−^) on each sample was directly influenced by the color of the nail polish (Table 2). The CCR sample, which showed the highest relative concentration, was light blue. Sudan III is known to be a red-to-brown dye under normal conditions, but when subjected to acidic conditions, it turns blue. This special dye also helps give a thick and shiny appearance, which was to be expected from the CCR sample. The other two samples with the highest content (RCR and ICR) are red to brown [47].

A comprehensive MS-based analytical methodology for the simultaneous screening of a large variety of coloring agents of great concern for regulatory control in cosmetics was established by Chen et al. [93] using ultra-high-performance liquid chromatography (UHPLC) coupled with quadrupole-orbitrap high-resolution mass spectrometry (Q-orbitrap HRMS) and ESI in positive- and negative-ionization modes. The chromatographic elution was performed with two binary mobile phase compositions for electrospray ionization in positive (ESI+) and negative (ESI−) modes, respectively. A 5 mmol/L ammonium formate solution at pH 5.5 and acetonitrile were paired for the ESI+ mode, and 5 mmol/L ammonium bicarbonate solution at pH 9.0 and acetonitrile were paired for the ESI− mode. The method was applied for the screening of the 63 coloring agents in 69 types of cosmetic samples (16 of lipsticks, 13 of eyeliners, 14 of blushers, 16 of eye shadows, 4 of toothpastes, and 6 of nail polishes), which were obtained from various sources.

In the initial stage, the cosmetic samples were subjected to the matrix solid-phase dispersion (MSPD) sample preparation method using anhydrous sodium sulfate and sand, then the MSPD column was further eluted with 2 mL of methanol and the extract was further analyzed by UHPLC-Q-orbitrap HRMS, under synchronous full-scan MS and data-dependent MS/MS (full-scan MS^1^/dd-MS^2^) acquisition mode. The identification and screening of target compounds were performed by using a self-built accurate-mass database and a customized mass spectral library and based on accurate mass agreement, consistency of retention time, characteristic ionic ratio, and isotopic distribution, the MS/MS spectra, and comparisons between theoretical and measured isotopic patterns [93].

Eleven legally prohibited coloring agents (Basic Violet 1, Basic Violet 10, Basic Violet 3, Pigment Red 3, Pigment Red 48:4, Solvent Blue 35, Solvent Red 23, Acid Orange 20, Pigment Red 48:2, Pigment Red 49, and Pigment Red 53:1) (Table 3) were detected in 26 cosmetic samples in total. In addition, some dye agents found in the studied samples were not labeled on their cosmetic packing.

The UHPLC Q-orbitrap HRMS method exhibited great potential for routine high-throughput, sensitive, and reliable screening of dye agents in cosmetic products for close quality control and to protect consumer health.

### 3.4. Analysis of Allergens in Cosmetic Products

Fragrances, next to heavy metals (nickel), preservatives, and hair dyes in cosmetics, are the most common cause of skin sensitization [94,95,96,97,98,99], which implies a life-long change in the immune system specificity. Skin allergy is clinically manifested as allergic contact dermatitis. Once sensitized, this condition rapidly develops upon re-exposure to a sufficient amount of the product containing the allergen. Based on several studies [99,100], perfumes, deodorants, and aftershaves were the riskiest product categories regarding sensitization and contact allergies. Moreover, in addition to contact dermatitis, other adverse effects like asthma, allergic rhinitis, migraine and photosensitivity, possible accumulation in the human body (associated with genotoxicity, which could lead to mutagenic or carcinogenic effects), and other side effects can also be developed.

Fragrances, such as perfumes and deodorants, aftershave products, shampoos, conditioners, laundry products, cleaning products, etc., are utilized in every aspect of our daily lives. Currently, more than 3000 chemical substances, either natural fragrance materials or synthetic fragrance chemicals, are responsible for odorous properties of scented products. At the same time, a mixture of 20 to over 200 constructs the fragrance compounds (including fragrance/aroma components, solvents, colorants, fixatives, and UV filters) [100,101].

Natural fragrances are divided into two major classes: aroma (obtained from plants/essential oils) and musk compounds (extracted from animal sources [102]). Because of the high prices of essential oils, dealers are tempted to adulterate the products by adding lower-cost materials, and, thus, synthetic aromatics can reduce perfume costs and are often used as an alternative source of compounds that are not easily obtained from natural sources or not found in nature.

Due to the adverse effects of fragrances, they are considered an emerging health and environmental concern [103]. For those substances responsible, or suspected to be responsible, for causing allergic reactions, their use has to be limited and/or strictly regulated. The International Fragrance Association (IFRA) periodically publishes a list of prohibited or restricted fragrance substances recognized by the IFRA expert panel [104]. In the EU, Regulation No. 1223/2009 [105] on cosmetics listed 26 fragrance ingredients, including two natural extracts (oak moss and tree moss) and 24 volatile chemicals that are considered to be more likely to cause allergic reactions in sensitive individuals (Table 4). These 26 fragrance substances are subject to specific labeling requirements if any individual concentration exceeds 10 µg/g for leave-on and 100 µg/g for rinse-off products. Such regulatory requirements necessitate consistent reference analytical methods suitable for routine quality control.

The Scientific Committee for Consumer Safety expanded, as of July 2023, the existing list of 26 regulated fragrance allergens with 56 new substances [106]. The contact allergens are classified as established, likely, and possible contact fragrance allergens. Among the 82 established contact allergens for humans (including the previous 26 regulated allergens), 28 are natural extracts and 54 are single chemicals. In order for cosmetic products to comply with the current legal requirements, the transition periods of three or five years must be taken into account.

The determination of fragrance substances in cosmetic products is challenging primarily because of the complexity of cosmetic formulations and the chemical similarity of the fragrance substances with other ingredients. This complexity makes it challenging to develop a universal method to cover all classes of cosmetic products [107].

Sample preparation is an essential step to remove interfering compounds and concentrate fragrance compounds in the sample before their analysis. Various preparation methods have been introduced, from direct analysis to methods with multiple clean-up steps. Liquid products (eau de toilettes and perfumes) are directly injected, without sample preparation, except dilution using different solvents or filtration [107,108,109] (if the amount of nonvolatile constituents is low when submitted to GC, as the performance of the GC system is rarely hampered by other constituents of the sample). If the analytes occur in more complex media, such as creams, lotions, foundations, and lipsticks, they need to be extracted from their matrix, prior to their analysis, via different established methods: by a fluid (in one step extraction technique [110,111,112,113] and improved variants such as MSPD: matrix solid-phase dispersion [23,93,114,115], QuEChERS (quick, easy, cheap, effective, rugged, and safe extraction) [116,117,118,119], or energy-assisted extraction techniques: vortex-assisted extraction (VAE) [120,121], ultrasound-assisted extraction (UAE) [121,122], and pressurized liquid extraction (PLE) [121,123], by liquid–liquid extraction (LLE) techniques [120,124,125,126,127,128], by solid-phase extraction techniques (μSPE: micro solid-phase extraction, dSPE: dispersive solid-phase extraction; SBSE: stir bar sorptive extraction) [129,130,131,132,133,134,135,136,137,138], and gas-phase extraction (headspace solid-phase microextraction (HS-SPME) [139,140,141,142,143].

MS, as a highly sensitive and selective analytical technique, has been used in qualitative and quantitative analysis of cosmetic products for ingredient screening and compound identification. Commonly, GC, as well as LC, hyphenated to MS or MS/MS were employed for routine evaluation of the analytes in different cosmetic matrices.

GC-MS is the most popular technique for fragrance analysis since they usually have low boiling points. Currently, GC is extensively used for sample infusion in EI-MS and has been greatly applied for the determination of allergens [28,31,98,107,144,145,146,147,148,149,150,151,152,153,154,155,156,157,158,159,160,161,162,163,164,165,166] and other risky components in cosmetic analysis (Table A1). In addition to EI, other ionization techniques like chemical ionization (CI) and photoionization (PI) are employed for GC-MS investigations in this field. Shibuta et al. [148] used multiphoton ionization (MPI) as the ion source by means of an fs laser, which emitted at 200 and 267 nm for the determination of 26 allergenic compounds in perfumes. This ion source proved suitable for the selective ionization of analytes by optimizing the wavelength of the light source, and the obtained limit of detection (LOD) values were all below 100 pg/μL. In fragrance analysis, GC was interfaced/coupled usually with quadrupoles (Q) [167,168,169,170,171,172,173,174,175,176,177,178,179,180,181,182,183,184], ion traps (IT) [153,157,185,186,187], and a few cases with time-of-flight (TOF) [148,188] mass analyzers, and in some situations, MS/MS analysis was also performed [107,189] for fragrance fingerprints. MS data acquisition was achieved typically in scan mode (full scan mode in different mass ranges) [190] selected ion monitoring (SIM)—in order to distinguish target fragrance, with known MS features and maximum sensitivity, making it ideal for quantification and validation [31,107,152,155,156,158,159,160,163,165,170,188], selected reaction monitoring (SRM)/multiple reaction monitoring (MRM) (resulting in increased selectivity, sensitivity, and signal/noise ratio [107,189], and selected ion storage (SIS), with a reduced LOD, due to the removal of parasite ions and more unrestricted spaces to store more ions of interests in the analyzer [162].

As the core problem for analyzing fragrance products is the large/abundant number of ingredients, up to several hundred different ones, and a broad concentration range, using a single column in the GC system could cause inadequate separation of analytes of interest [28]. In this view, to improve selectivity in complex cosmetic matrices, several methodologies based on multidimensional comprehensive approaches have been developed by a few working groups [107,109,167,170,173,174,191], and the recent recommendation [192] is to use 2D GC systems for fragrance investigation to avoid obtaining negative false or positive results.

LC-MS is used in the analysis of fragrance components that are challenging to analyze by GC, because of their decreased volatility and/or their thermostability (Table A1, [193,194]). C18 columns with various characteristics were generally used [195,196,197,198], while in some particular situations, C8 columns were preferred [199]. The use of a single mobile phase hampered the separation of all components of the multicomplex formulation of cosmetics, making it necessary to use chromatography with various gradient profiles [195,197].

ESI, atmospheric pressure chemical ionization (APCI), and atmospheric pressure photoionization (APPI) are ionization procedures frequently coupled with LC-MS systems. Mainly, ESI shows good versatility in the ionization of various substances, and LC ESI-MS is quite ideal for the analysis of large, fragile molecules [200]. In contrast, APCI is usually used for weakly polar compounds with a mass below 1500 Da [201], and APPI is more appropriate for nonpolar compounds that are difficult to charge [202]. Single (Q) [195] or triple (3Q) quadrupoles [196,197] are primarily used in conjunction with LC-MS in the determination of fragrance allergens. MS data were acquired in full scan and SIM modes [195], as well as in SRM mode in other experiments [197].

Convergence chromatography (CC), a separation technique used to bridge LC and GC, uses carbon dioxide as the primary mobile phase with the option (if necessary) of using an additional cosolvent, such as acetonitrile or methanol, to obtain selectivity similar to that of the normal phase LC. This method was developed as ultra-performance convergence chromatography (UPC^2^) with MS detection for the analysis of scented allergens in perfumes, cosmetics, and personal care products in a very short time of 7 min [203] (Table A1). It is an ideal alternative to both HPLC and GC analysis, offering a few advantages such as the following:

(a) The ability to investigate compounds suitable for LC and GC in a single analysis;
(b) A higher selectivity and specificity compared to HPLC or GC analysis only;
(c) An analysis time at least six times faster than for HPLC and GC;
(d) Solvent use is 95% lower than in existing HPLC methods.

Because of the challenging sample preparation and chromatographic separation, GC- and LC-MS-based methods cannot provide rapid and high-throughput analysis. Thus, the development of advanced ionization techniques like ambient ionization mass spectrometry (AIMS) offers simplicity, reproducibility, and efficiency in cosmetic analysis, but its usage in allergen identification is scarce to date. Liu et al. [204] used dielectric barrier discharge (DBD)-MS to detect the presence of fragrance allergens in commercially available perfume products, obtaining a reasonable linear range and low LOD, sometimes at ppt levels. DBD ion sources contain very few components (two electrodes, one a stainless-steel needle or wire and the other a copper strip separated by an insulating barrier such as glass, between which, upon application of a high voltage, a low-temperature plasma form that can be applied directly to the surface of liquid or solid samples, or into which gas state samples can be introduced) and can be created very economically compared to commercialized sources, and has been shown to be a potent instrument in detecting airborne allergens [205].

The MS technique can be used alone to explore fragrance fingerprints, essential for counterfeiting discovery. Marques et al. [206] used direct infusion by ESI of perfume samples (original, counterfeit, and “inspired”) diluted by methanol/water (1:1) in Q-TOF-MS, and the data were acquired in positive-ion mode. The peak lists were analyzed further by principal component analysis (PCA) so that ESI-MS fingerprinting allowed for the fast and reliable detection of perfume falsification. Quite similar profiles in positive-ion mode were obtained by Haddad et al. [207] by easy ambient sonic-spray ionization (EASI) Q-Trap MS and PCA analysis for counterfeit recognition of perfume samples, while Chingin et al. [208] used ESI-Q-TOF-MS in positive-ion mode without PCA analysis for rapid fingerprinting of various perfumes and forgery identification. In the above-presented reports, many *m*/*z* peaks detected with considerably higher signal levels in MS spectra from the counterfeit perfume are attributable to the low-purity materials used for their production, which can be responsible for some known side effects as allergies and toxicity.

Identification of fragrance compounds based on their relative retention times in GC, as well as their mass spectra, depends directly on the quality and comprehensiveness of the library used. Only libraries of EI mass spectra are efficient, and other ionization techniques yield spectra that are much too dependent on the instruments and experimental conditions [155]. The most common mass library is National Institute of Standards and Technology (NIST), composed of three primary parts: the first part is “NIST/EPA/NIH Mass Spectral Library (EI)” and contains “Main EI MS Library” and “Replicate EI MS Library”; the second part is “Tandem (MS/MS) Library”, which comprises small molecules and biologically-active peptides sections; and the last one is titled “GC Method/Retention Index Library” [209]. This library is typically used in the fragrance industry, e.g., various research groups [140,174,181,182,185] identified different suspected fragrance allergens by comparison of the experimental spectra with those of the NIST database. Also, Wiley, National Bureau of Standards library, was prepared by McLafferty et al., in which the registry spectra for 108,173 compounds are available [155]. This library was also used for qualitative analysis of fragrances in cosmetics and perfumes [140,168,174,185]. The main disadvantage of such libraries is the inability to distinguish between compounds with identical mass spectra due to the lack of retention data. While the retention times vary with the different chromatographic system, the retention index is a system-independent constant calculated by a formula that varies with the polarity of a column and is used in certain libraries, which makes them more reliable. For example, the “Flavour & Fragrance Natural & Synthetic Compounds” (FFNSC) GC-MS library, prepared by the group of Mondello et al. [28], contains retention indexes compatible with three types of columns, including nonpolar column (EquityTM-1), micropolar column (SLBTM-5 ms), and highly polar column (SUPELCOWAXTM 10). Mondello and coworkers identified the allergens by using the FFNSC mass spectral library database [28]. The third edition of this library (FFNSC 3) is registered with 3462 natural and synthetic chemical compounds relating to flavors. Additionally, Tranchida et al. employed this library to detect the recently highlighted fragrance allergens (54) in cosmetics (by the Scientific Committee on Consumer Safety) [107].

Other libraries, like Adams and MassFinder, are dedicated to essential oils and comprise the retention index of each compound measured on a nonpolar column [155]. In some situations, when analysis of fragrance is a major activity, the best method is to build a home library, e.g., the in-house fragrance MS library, which was built on an HP Chemstation platform by Liu et al. [187], and another example is the in-house “Baser Library of Essential Oil Constituents”, which contains MS and retention data of over 3500 genuine compounds found in essential oils [155].

Another custom-made accurate-mass database was constructed for the comprehensive identification of 100 multiclass regulated ingredients in cosmetics [209]. The cosmetic samples were analyzed by UHPLC Q-orbitrap HRMS, using individual standard solutions of analytes at a concentration of 100 ng/mL, under synchronous full-scan MS and data-dependent MS/MS (full-scan MS1/dd-MS2) acquisition mode. The custom-made accurate-mass database was made by inputting the exact mass information of the 100 target compounds and their characteristic fragments together with their respective chemical name, molecular formula, and chromatographic retention time using a TraceFinder version 4.1 software supplied with the instrument (Thermo Fisher Scientific, Waltham, MA, USA). In addition to the accurate-mass database, a mass spectral library was built by importing the acquired mass spectra containing both precursor ions and all characteristic fragment ions for the 100 target compounds using the TraceFinder software. The mass spectral library was employed in combination with the accurate-mass database to search and identify the target compounds of interest from the UHPLC-Q-orbitrap HRMS raw data based on retention time, precursor and MS/MS fragment ions, ionic ratio, isotope pattern, and mass accuracy tolerance [209].

### 3.5. Analysis of Heavy Metals in Cosmetic Products

Since cosmetic products are complex mixtures of texture and coloring agents, a variety of metals can be present in them. Heavy metals can occasionally be found as contaminants in cosmetic products (Table 5) due to impurities in raw materials or the manufacturing procedure, processing aids, and contamination in the supply chain. Even if their presence as impurities in cosmetic products is inevitable due to the ubiquitous nature of these elements, they should be removed wherever technically possible. Thus, the regulatory agencies (Food and Drug Administration (FDA), Federal Office of Consumer Protection and Food Safety (BVL), etc.) make efforts to establish limits on the levels of heavy metals in cosmetics, varying by product and country. Nevertheless, even the very perilous heavy metals are often not explicitly regulated or entirely banned. Guidance on the safety assessment of cosmetic ingredients has been published by the EU Scientific Committee on Consumers Products (SCCS, 2012). The Annex II (“List of substances which must not form part of the composition of cosmetic products”—[210]) of the Directive lists more than 1000 chemical substances that cannot be used in cosmetic products due to their toxicological properties. According to this Annex, several metals, such as antimony (Sb), arsenic (As), cadmium (Cd), chromium (Cr), cobalt (Co), mercury (Hg), nickel (Ni), and lead (Pb), are prohibited ingredients in cosmetics because they are considered unsafe and are designated as having toxic and/or allergological concern. However, there are currently no international standards for heavy metal impurities in cosmetics. Limits for some dangerous heavy metals in cosmetics have been established in Germany and USA (Table 6, [211,212]), but also in other countries worldwide [213,214,215,216,217].

The meticulous analysis of heavy metal compounds found in cosmetics holds paramount importance due to the potential health risks associated with exposure to these elements (Table 6). Despite abundant research on metal detection in cosmetic products [218,219,220,221,222,223,224,225,226,227,228], very few of these studies focused on estimating human exposures and health risks from cosmetics, highlighting the need for comprehensive risk assessments.

**Table 6:** The permissible limits of heavy metals in cosmetic products and their adverse health effects.

No.	Heavy Metal	Limits for Cosmetics (EU, Germany)	Limits for Cosmetics (USA)	Effects of Exposure on the Human Body
1	Mercury (Hg)	0.1 ppm *	1 ppm (colorants)	Renal, neurologic, and dermal toxicity [229], cutaneous changes reported include burning of the face, contact dermatitis, grey or blue–black facial discoloration, flushing, erythroderma, purpura, and gingivostomatitis.
2	Lead (Pb)	2 ppm	20 ppm (colorants) 10 ppm (lipsticks, lip glosses)	Affects the fetus and the central nervous system in Children [230,231], probably carcinogenic to humans [232,233], neurotoxic, nephrotoxic, and hepatotoxic and can also produce effects on the reproductive system, and can also affect fetal development through its passage via the placenta [234,235,236,237,238,239].
3	Cadmium (Cd)	0.1 ppm	-	Damage of the kidneys, fragility of the bones, carcinogenic in humans [240,241,242].
4	Arsenic (As)	0.5 ppm	3 ppm (colorants)	Skin eruptions, alopecia, and striation of the nails, but also skin cancer [243], circulatory and peripheral nervous disorders, an increased risk of lung cancer, and a possible increase in the risk of gastrointestinal tract and the urinary system cancers [244].
5	Nickel (Ni)	10 ppm	-	Contact allergy, eyelid dermatitis, as well as irritation, eczema
6	Chromium (Cr)	-	-	contact allergy [245], carcinogenic in humans (Cr(VI)).
7	Antimony (Sb)	0.5 ppm	-	Pneumoconiosis, alterations in pulmonary function, bronchitis, emphysema, gastrointestinal effects (abdominal pain, vomiting, diarrhea, and ulcers), dermatoses, and skin lesions [246,247,248], probably carcinogenic to humans (Sb trioxide).
8	Cobalt (Co)	-	-	Skin allergen causing allergic contact dermatitis (ACD) and eczema, possibly carcinogenic to humans.

Note: * ppm—parts per million.

Employing advanced techniques like MS for accurate determination allows for the identification and quantification of trace amounts of heavy metals in cosmetics. This not only ensures compliance with regulatory standards but also helps in establishing a robust framework for consumer protection. The significance of such analyses becomes particularly pronounced given the widespread use of cosmetics and the potential impact on diverse consumer demographics, emphasizing the need for stringent quality control measures in the cosmetic industry.

Inductively coupled plasma mass spectrometry (ICP-MS) is a powerful analytical technique widely used for determining the presence of heavy metals in various substances, including cosmetics. In ICP-MS, a high-temperature plasma torch is used to ionize the sample, creating charged particles, which are further sorted and measured based on their *m*/*z* ratio. The ionization process is considered a ”hard process”, unlike other MS techniques, such as ESI-MS, which is considered a ”soft” ionization technique [249].

ICP-MS is incredibly useful in cosmetics testing because it provides highly sensitive and precise results, even at trace levels of heavy metals. Its utility lies in its ability to detect and quantify multiple elements simultaneously, offering a comprehensive analysis of the sample composition. This makes ICP-MS an essential tool for ensuring the safety and compliance of cosmetics, as it helps identify potential contaminants and ensures that products meet regulatory standards, safeguarding consumer wellbeing [250]. ICP-MS was used in many studies related to heavy metal analysis in cosmetics and proved to be a good choice for determining low analyte concentrations, thus allowing the evaluation of toxic and potentially toxic components in cosmetics [222,223,224,225,226,228,251,252,253,254,255,256,257,258,259].

Grosser et al. [228] showed that the levels of several heavy metals (Pb, As, Cd, Hg, Cr, Se, Sb) in cosmetics such as lipsticks, nail polishes, and skin creams could be determined by ICP-MS after preparation of the samples by microwave digestion in order to obtain clear solutions (except for the determination of Hg) and the obtained results indicated values below the risk limits according to Canadian regulations.

Bobaker et al. [253] studied traditional plant-based beauty products marketed in Libya, respectively, henna (derived from the *Lawsonia inermis*) and walnut tree bark (also known as souak—used both as a dental care product and as a popular enhancer for henna colors) and raised concerns about the potential presence of heavy metals such as Pb, Cd, and As in these cosmetics, posing health risks (due to their narrow safety margins) through skin absorption, and their accumulation in internal organs, which can result in toxicity. Before heavy metal analysis by ICP-MS, the samples were processed (walnut tree bark samples), oven-dried, and subjected to microwave-assisted digestion after the addition of a nitric acid (HNO_3_)/hydrogen peroxide (H_2_O_2_) mixture [260,261,262]. ICP-MS analysis (Perkin Elmer SCIEX Elan 9000 ICP-MS, Perkin-Elmer SCIEX, Norwalk, CT, USA) of the samples revealed the presence of Pb, Cd, and As, with mean concentrations (Pb > Cd > As) having abnormally high standard deviations, which can be attributed to differences in product manufacturing, contamination levels, and the diverse sources of the products, influenced by varying climatic conditions. Walnut tree bark samples exhibited higher heavy metal levels compared to henna samples, and higher Pb concentrations were observed in black henna compared to green henna samples. Additionally, most henna and walnut tree bark products exceeded recommended limits for Pb and Cd, while As levels were generally lower.

Rubio et al. [254] conducted similar research on henna and jagua traditional cosmetic products, whose composition can be influenced by several factors, including post-processing and contamination. The metal composition (including 11 elements, i.e., Al, Cu, Zn, Ba, Mn, Co, Ni, Pb, Cr, Cd, and As) of henna and jagua commercial samples was investigated using ICP-MS (Agilent 8800 Triple Quadrupole(QQQ) ICP-MS device, Agilent Technologies, Hachioji, Tokyo, Japan), equipped with a MicroMist nebulizer and a quartz spray chamber, after initial steps of predigestion and digestion, using HNO_3_ and H_2_O_2_ solutions. Several henna samples showed high concentrations of Mn, Zn, and Pb, while Cu and Cr levels were elevated in certain jagua samples. Banned elements, including Cr, Ni, As, Cd, Co, and Pb, were found in trace amounts, raising concerns about unintentional contamination. The method demonstrated strong linearity coefficients, accuracy, and precision in determining heavy metal content in henna and jagua samples.

The impact of heavy metals present in homemade traditional cosmetic products (lipstick, lip moisturizer, eye mascara, eyeliner, henna, tattoo, and spray hair dye) on human and environmental health was investigated by Killic et al. [255]. The study used ICP-MS on an Elan DRC-e (Perkin Elmer SCIEX, Norwalk, CT, USA) to assess the metal content in homemade cosmetic products, comparing them to WHO’s (World Health Organization) permissible limits. This research involved the collection of cosmetic products from the market, with hair spray dye serving as a matrix for method validations. The samples collected from the market were subjected to microwave digestion using HNO_3_ and H_2_O_2_ digestion, followed by ICP-MS analysis of the diluted samples in order to determine As, Cd, Co, Cr, Cu, Ni, and Pb levels. The optimized methods have proven high selectivity and sensitivity, with detection limits LOD of 0.1–0.2 μg/L and LOQ of 0.2–0.8 μg/L for metal assessment. Pb was found in all samples, surpassing the WHO-specified range, while As, Co, Cd, Cu, Cr, and Ni were present in variable concentrations in certain products. The average heavy metal levels differed across cosmetic categories, with Pb being constantly identified. Pb and Ni concentrations in the smear exceeded the permissible WHO limits, while Pb concentrations in other products were below the limit. Metal levels in spray hair dye, smear, and tattoo usually remain at trace levels. The health risk was evaluated using the target hazard quotient (THQ), which is the ratio of exposure to the toxic element to the dose at which adverse health effects are expected to occur. The THQ index for lipstick showed varying values, in descending order of Cr > Pb > Ni, while for other cosmetics, it was Pb > Cr > Ni. The recommendation is that despite THQ values being lower than 1 for all tested samples, which will not result in immediate health risks, persistent and excessive use of these products could possibly lead to long-term health problems for users.

Salama et al. [251] focused on evaluating the concentrations of 10 heavy metals (Pb, Al, Cd, Co, Cr, Cu, Mn, Ni, Hg, and As) in various cosmetic products (beauty cream, hair cream, skin cream, hair gel, hair food formula, etc.) available in the Saudi Arabian market using ICP-MS (NexION 300 D, Perkin-Elmer, Inc., Shelton, CT, USA) method. Before MS analysis, cosmetic samples were processed by dry-ashing method, but for particular samples like creams and lotions, wet digestion using HNO_3_ and HClO₄ was accomplished. The results indicated that the levels of heavy metals varied considerably across different product types: in shampoo products, the highest mean concentration was obtained for Al, followed by Pb, Cu, Cr, Mn, Ni, Hg, As, Co, and Cd; in cream products, the highest average concentrations were for Al, followed by Cu, Mn, Pb, Cr, Ni, Hg, Co, As, and Cd; while soaps exhibited the highest mean levels for Al, followed by Cu, Pb, Cr, Mn, Ni, As, Co, Hg, and Cd; and toothpaste products displayed the highest mean levels for Al, followed by Cu, Mn, Pb, Cr, Co, Ni, Cd, As, and Hg. Overall, Al was shown to be the major heavy metal in most samples, with exceptions in several skin, beauty, and shaving creams. Cu followed Al in concentration, except in certain shampoo products, and Cd was identified as having the lowest levels, except in toothpaste, where it was replaced by Hg for position in ranking.

Perez et al. [256] investigated the exposure (via incidental ingestion and via dermal uptake) to metals in costume cosmetics for three subject categories: child (2–3 years), adult (≥18 years) with infrequent use (12 times per year), and adult with daily occupational use. Costume cosmetic products in different forms (lotion, spray, stick, eyeshadow), containing both water-based and oil-based commercial costume makeup, were prescreened for metal content using a handheld X-ray fluorescence (XRF) detector, and those with detectable metals were subjected to a concentrated HF/HCl/HNO_3_ digestion and analyzed further by ICP-MS (Agilent 8800) using a triple quadrupole analyzer (ICP-QQQ-MS). A SkinPerm absorption model was employed to estimate dermal exposure concentrations and absorbed doses, while to evaluate possible metal exposure via incidental ingestion, the measured concentrations were used to calculate the potential exposure from direct ingestion (i.e., from lipstick) or from ingestion via hand-to-mouth contact, using exposure assumptions, many of which are based on the statistical data [263]. The daily intake doses from oral exposure were estimated using the method described in [264]. The results showed the presence of Sb, Pb, Ni, Co, Cr, Hg, and Ag from below detection to 9.3 mg/kg wet weight in various samples. This was found in one body paint sample, while Cd and methylmercury (MeHg) were not detected. Oral ingestion accounted for over 99% of all metal intake. The Pb dose from body paint was predicted to raise blood lead levels above baseline in all users, with an increase of less than 1 µg/dL amongst the child and adult-intermittent users, while occupational usage raised blood lead levels by 1.0 and 1.9 µg/dL for mean and maximum Pb concentrations, respectively. Overall, the study revealed that costume cosmetics with varying levels of As, Co, Ni, Pb, and Sb do not pose unnecessary health risks to intermittent consumers; however, occupational exposures may exceed recognized health-based control values.

Similarly, Salles et al. [257] focused on the presence of metallic-based pigments, including heavy metals in liquid, cream, or pancake face paints, of various colors, commonly applied to the head and trunk, with specific attention to occupational exposure (in adults) and children’s vulnerability (related to costume cosmetics) in Brazil. A set of potentially toxic elements (Al, As, Ba, Cd, Co, Cr, Cu, Ni, Pb, Sb, Sn, Sr) was determined through HNO_3_ digestion, followed by ICP-MS analysis (Agilent 7900, Hachioji, Japan). The dermal exposure evaluation involved calculating cancer risk and dermal hazard quotient based on dermal absorption during product use. Incidental ingestion exposure assessment estimated potential exposure via hand-to-mouth contact.

The study revealed statistically significant differences in sample concentrations between colors, especially for As, Ba, Cd, Co, Cu, and Pb, with variations in mean concentrations, i.e., white, brown, and lilac paints showed elevated mean levels for As, red colors for Ba, and white, purple, and blue displayed the highest means for Pb and Cd. Yellow and brown colors had the highest average concentrations for Co, while lilac, blue, and green colors showed the highest means for Cu.

In general, for nearly all elements, pancakes and liquid samples had higher means. Cream samples and professional pancakes had higher average concentrations for Cd, Cr, and Pb, while those of Sr were higher in fluorescent and liquid paints. The levels of Cu did not differ between types of costume cosmetics. High cancer risk was identified in both children and occupational exposures to potentially toxic elements in costume cosmetics. For children, the risks from accidental ingestion exceed those from dermal exposure due to hand-to-mouth behavior. In contrast, for adults, the risk is higher through dermal exposure, highlighting the need to monitor these elements in cosmetic products globally to safeguard human health, particularly for continuous professional use and child consumption during festive events.

Domeradzka-Gajda et al. [258] used ICP-MS to explore the impact of various cosmetic ingredients, including parabens (methylparaben), phthalates (dibutyl phthalate), and (aluminum salts AlCl_3_) on the percutaneous absorption of silver nanoparticles (AgNP) using an in vitro model based on isolated pig skin. AgNP, the most prominently advertised nanomaterial, is present in numerous consumer products, including cosmetics, personal care, health, and food items. The Nanodatabase and ANEC/BEUC inventory [265] list numerous nanosilver-containing products related to skincare: crèmes, moisturizers, washing lotions, cleansers, antiperspirants, soaps, shoe deodorants, or foot balms. The majority of AgNP applications results from their strong antibacterial properties [266] primarily linked to the release of Ag ions, but their percutaneous absorption is a subject of concern. Studies on medical products containing AgNP indicate possible skin penetration, especially at the level of injured skin [267,268,269,270].

Scarce research exists on the percutaneous absorption of AgNP through normal skin, but evidence suggests detectable penetration [271,272,273]. The absorption may vary when AgNP interacts with other cosmetic ingredients, especially on microabraded skin.

The authors used, for the purpose of the study, AgNP of different conventional sizes (15 nm or 45 nm) and surface modification, and as citrate or PEG stabilized nanoparticles and various matrices—pig skin sections and receptor fluid (Franz chambers). The determination of Ag content in receptor fluid was conducted by ICP-MS using a PerkinElmer Elan1 DRC-e device, Perkin Elmer, SCIEX, Framingham, MA, USA. The sample introduction system included a quartz cyclonic spray chamber, Mainhard nebulizer, and a peristaltic four-channel pump. Triplicate measurements were performed for each sample, with deionized water analyzed between replicates to check for memory effects. For skin samples, laser ablation (LA)-ICP MS was applied by using skin disks, post 24 h exposure in the Franz chambers, washed and fixed in 10% neutral buffered formalin, embedded in paraffin, and sectioned at 20 mm. LA-ICP-MS data were processed into 2D elemental images using Applied Spectra, Inc.’s ((West Sacramento, CA, USA) Data Analysis Software (http://www.appliedspectra.com/downloads.html).

ICP-MS measurements after 24 h in receptor fluid indicated low but detectable silver absorption and no correlation with concentration, nanoparticle size, or the mode of nanoparticle stabilization. Furthermore, the chosen cosmetic ingredients (methylparaben, dibutyl phthalate, and AlCl_3_) did not exert a statistically significant influence on silver absorption, with the highest amount of Ag that penetrated (0.45 ng/cm^2^) being measured, such as for PEG stabilized Ag of 15 nm + methylparaben.

In addition to many studies that are focused on trace metal analyses in cosmetic matrices like colored make-up products (as a lot of pigments contain metal compounds), Rujido-Santos et al. [259] analyzed the metal content in the most commonly used moisturizing creams on the Spanish market, to verify their degree of compliance with [274] regarding the presence of metals in cosmetics, as they have various functions in formulations of these cosmetic products [275]. The study used a validated methodology based on microwave-assisted acid digestion of the samples followed by ICP-MS and showed that most of the analyzed products did not comply with Regulation (EC) No. 1223/2009 [276] on cosmetic products, while for several cases, speciation studies were necessary. Only one moisturizing cream fulfilled the stated regulation.

## 4. Conclusions

Cosmetics and personal care products are complex formulations comprising a vast number of ingredients with diverse physicochemical properties. As a primary concern, cosmetic products that are commercially available must be safe for users. Providing cosmetics safety and implementing compliance with standards, regulatory requirements, and quality management systems requires efficient and robust analytical methods. Recent advances in MS have contributed to many scientific findings in the cosmetic industry by investigating very complex mixtures of chemical ingredients and their interactions with the human body. Still, many directions have not yet been examined. Analysis of bioconjugates in cosmetics by MS-based methods is a narrow field, mainly limited to the analysis of different lipids, including ceramides, for the quality control of the product formulation by MALDI-MS and ESI-MS, respectively, and CID used to carry out the fragmentation of analytes. Investigation of dextrins in dermal cosmetics is relatively unexplored, and in this area, MALDI-MS proved suitable for the characterization of their chemical structure in order to determine their prospective applications. Both LC (HPLC, UHPLC)- and GC-MS (which commonly requires derivatization of the analytes) are widely used for the quantification of preservatives in cosmetics and personal care products, especially in the case of counterfeit cosmetics, as they are unregulated and may contain levels of preservatives that can lead to health risks associated with long-term exposure. Given the increased selectivity and sensitivity, MS hyphenated to different LC variants (LC, HPLC, and UHPLC) is the usual choice for accurately identifying and quantifying dyes in cosmetic samples due to their hydrophilic nature, especially in the analysis of banned compounds. In addition, ambient ionization techniques such as DESI interfaced to MS have been used for the direct analysis of the sample without prior preparation or chromatographic separation, even for personal care products directly on the cell surface, while MALDI-MSI usage in this direction was limited to the analysis of dyes in nail polish samples.

Moreover, the input of HR detectors allowed comprehensive UHPLC-ESI-HRMS simultaneous screening of a great diversity of coloring agents of significant concern for regulatory control in cosmetics. Usually, GC as well as LC coupled to MS or MS/MS and using diverse ion sources were employed for routine evaluation of analytes having allergenic properties in different cosmetic matrices, mainly perfumes. Since fragrance products consist of a large number of ingredients, methodologies based on multidimensional GC-MS approaches are preferred by many researchers. With the exception of perfumes, the investigations of most cosmetic matrices imply steps of sample preparation and chromatographic separation in advance and, thus, cannot provide rapid and high-throughput analysis. Ambient ionization approaches (AIMS) yield efficiency, reproducibility, and simplicity in cosmetic analysis, but their utilization in allergen determination has been scarce up to the present. The introduction of convergence chromatography (UPC^2^) with MS detection has been proven to be an excellent choice in addition to HPLC and GC for the analysis of scented allergens because of its ability to investigate compounds suitable for LC and GC in a single analysis, with a reduced volume of the used solvent, and of good analysis time. “Hard ionization” ICP-MS preceded by microwave-assisted digestion is ideal for detecting and quantifying multiple elements simultaneously, offering a comprehensive analysis of heavy metals present in different samples and being able to evaluate human exposure and possible health impacts. In the future, there is no doubt that MS-based methodologies will continue to be improved to meet the increasing complexity of regulatory constraints in the cosmetic field, and expanding their use will undoubtedly increase the efficiency of cosmetic quality and safety assessment to a significant level.

## Figures and Tables

**Table 1:** The parabens detected and quantified in hair samples by LC-MS/MS.

Paraben	Monoisotopic Mass (Da)	Concentration (ng/g, Mean ± SD)	Range (ng/g, Mean ± SD)
MP	152.047348	123.6 ± 61.6	48.3–224.2
EP	166.062988	64.5 ± 43.5	11.5–158.3
PP	180.078644	136.9 ± 48.5	70.2–214.5
BP	194.094299	74.2 ± 27.5	25.4–111.1
BeP	228.078644	55.6 ± 24.3	15.3–100.2

**Table 2:** Nail polish samples and the color for each. The third column refers to the aspect of each product.

Sample Nail Polish	Color	Aspect
AHC	Dark blue	Creamy
CCR	Light blue	Creamy–shiny
HTT	Brown	Simple
ICM	Red	Metallic
ICR	Orange-Golden	Creamy
IPC	Orange	Simple
RCR	Red	Creamy
RMT	Pink	Metallic
SLB	Golden	Simple

**Table 3:** Legally prohibited coloring agents detected in 26 cosmetic samples [93].

No.	Dye Agent	Molecular Ion (*m*/*z*)	Molecular Formula	Structural Formula
1	Basic Violet 1	358.22696	C_24_H_28_N_3_	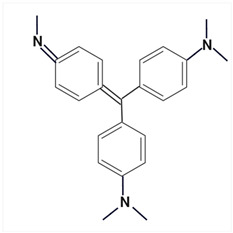
2	Basic Violet 10	443.23227	C_28_H_31_N_2_O_3_	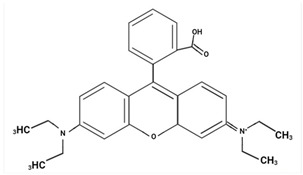
3	Basic Violet 3	372.24277	C_25_H_30_N_3_	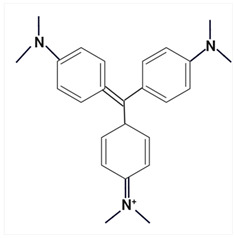
4	Pigment Red 3	308.10236	C_17_H_13_N_3_O_3_	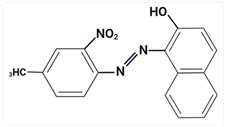
5	Pigment Red 48:4	421.02478	C_18_H_13_ClN_2_O_6_S	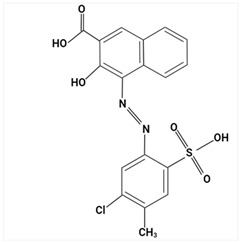
6	Solvent Blue 35	351.20642	C_22_H_26_N_2_O_2_	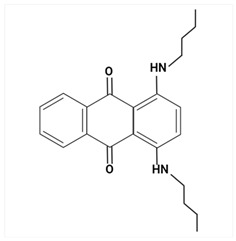
7	Solvent Red 23	353.13892	C_22_H_16_N_4_O	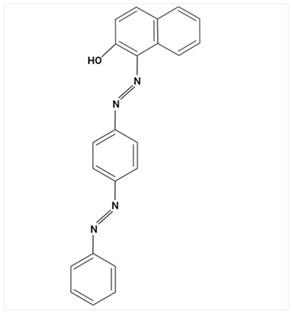
8	Acid Orange 20	327.0441 [M − Na^+^]^−^	C_16_H_11_N_2_O_4_S	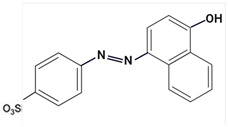
9	Pigment Red 48:2	419.01062	C_18_H_11_CClN_2_O_6_S	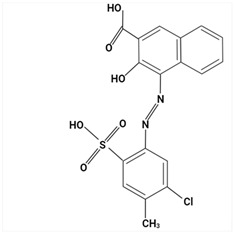
10	Pigment Red 49	377.05936	C_20_H_14_N_2_O_4_S	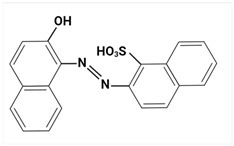
11	Pigment Red 53:1	375.02057		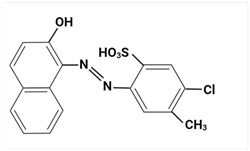

**Table 4:** Chemical characterization of 24 individual fragrance ingredients associated with allergic reactions (according to EU, Regulation No. 1223/2009 [105]).

No.	Fragrance Allergen	Molecular Formula	MW
1	Alpha isomethylionone	C_14_H_22_O	206.32
2	Amyl cinnamal (Jasmonal A)	C_14_H_18_O	202.29
3	Amyl cinnamyl alcohol	C_14_H_20_O	204.31
4	Anisyl alcohol	C_8_H_10_O_2_	138.16
5	Benzyl alcohol	C_7_H_8_O	108.14
6	Benzyl benzoate	C_14_H_12_O_2_	212.24
7	Benzyl cinnamate	C_16_H_14_O_2_	238.28
8	Benzyl salicylate	C_14_H_12_O_3_	228.24
9	Butylphenyl methylpropional (Lilial)	C_14_H_20_O	204.31
10	Cinnamal	C_9_H_8_O	132.16
11	Cinnamyl alcohol	C_9_H_10_O	134.17
12	Citral	C_10_H_16_O	152.24
13	Citronellol	C_10_H_20_O	156.26
14	Coumarin	C_9_H_6_O_2_	146.14
15	Eugenol	C_10_H_12_O_2_	164.20
16	Farnesol	C_15_H_26_O	222.37
17	Geraniol	C_10_H_18_O	154.25
18	Hexyl cinnamal (Jasmonal h)	C_15_H_20_O	216.32
19	Hydroxycitronellal	C_10_H_20_O_2_	172.26
20	Hydroxyisohexyl 3-cyclohexene carboxaldehyde (Lyral)	C_13_H_22_O_2_	210.31
21	Isoeugenol	C_10_H_12_O_2_	164.20
22	Limonene	C_10_H_16_	136.23
23	Linalool	C_10_H_18_O	154.25
24	Methyl 2-octynoate	C_9_H_14_O_2_	154.21

**Table 5:** Heavy metals contaminants in cosmetic products.

No.	Heavy Metal	Cosmetic Products
1	Mercury (Hg)	Creams (antiseptic, skin-lightening) and some mascaras
2	Lead (Pb)	Lipsticks, eyeliners, lip glosses, and hair dyes
3	Cadmium (Cd)	Blush, eyeshadow, and face powders
4	Arsenic (As)	Blush, eyeshadow, and face powders
5	Nickel (Ni)	Foundations, eyeshadow, and mascaras
6	Chromium (Cr)	Eyeshadow, lipsticks, and face powders
7	Aluminum (Al)	Foundations, eyeshadow, and mascaras
8	Copper (Cu)	Blush, eyeshadow, and some lipsticks
9	Antimony (Sb)	Blush, eyeshadow, and mascara
10	Zinc (Zn)	Foundations, sunscreens, and face powders
11	Manganese (Mn)	Blush, eyeshadow, and some lipsticks
12	Cobalt (Co)	Hair dyes and some eyeshadows
13	Selenium (Se)	Hair dyes and some face powders
14	Barium (Ba)	Blush, eyeshadow, and face powders
15	Beryllium (Be)	Eyeshadow and face powders
16	Thallium (Tl)	Hair dyes and some face powders

## Data Availability

No new data were created or analyzed in this study. Data sharing is not applicable to this article.

## Appendix A

**Table A1:** MS-based analysis of allergens in cosmetic products.

No.	Samples	MS-Based Analysis	Observations/ Comments	Ref.
1	A total of 42 cosmetic products (12 rinse-off and 30 leave-on) purchased from nine different countries (USA and EU).	GC-MS	Simultaneous determination of 30 fragrances (including 24 listed allergens)—18 leave-on products contained at least one fragrance substance > 10 µg/g and 5 of the 12 rinse-off products contained at least one fragrance substance > 100 µg/g.	[166]
2	A total of 166 leave-on cosmetic products purchased within three years and stored at room temperature.	GC-MS and GC-MS/MS	The method was tested in order to identify the presence of 24 regulated allergens and 21 prohibited substances in the cosmetic products: 2–17 allergens were identified per sample, and only safrole (of the prohibited substances) was present in a concentration > LOQ in 12 out of 166 tested samples.	[144]
3	Six fragrance compositions.	GC HR-MS and GC-LR-MS	A total of 35 “difficult fragrance allergens” were quantified best by GC-orbitrap HR-MS.	[145]
4	A “Lily” matrix—a combination of about 50 raw materials, mainly constituted of essential oils, aromatic plant extracts, synthetic ingredients, water and ethanol.	GC-qMS	The validation of the GC-MS method for the quantification of the extended list of 57 fragrance allergens (which led to the updated NF EN 16274 standard in 2021 [276]).	[146]
5	One tested fragrance (CC02).	GC×GC qMS (SIM mode)	Validating a method to identify and quantify the suspected allergens (24) limited by EU regulations in fragrances by GC×GC-qMS.	[109]
6	Initial screening of 5 cosmetics (3 creams and 2 lotions) and subsequent screening of 123 cosmetics (71 creams and 52 lotions).	UHPLC-q-orbitrap HR-MS	Simultaneous screening of 100 restricted ingredients in cosmetics (39 antibodies, 40 glucocorticoids, 9 androgens, 8 progestogens, 4 antifungal agents.	[209]
7	Two commercial perfumes (“eau de toilette”).	GC–EI-MS	The 1460 and 1910 Watercol^TM^ columns can reliably be used for the GC-MS analysis of EU-regulated volatile allergens in commercial perfumes (14 allergens identified in perfume 1 and 4 allergens in perfume 2) and showed complementary selectivity.	[150]
8	Three commercially available perfumes (Sakura Eau de Toilette, Moroccan Rose, White Musk).	GC-MPI-TOF-MS	A total of 26 allergens were identified (superior performance of MPI/MS over EI/MS for more reliable determination of the allergy compounds); the concentrations of methyl-2-octynoate (not written in the label of the bottle), citronellol, hexylcinnamaldehyde, and linalool in Sakura Eau de Toilette, and methyl-2-octynoate in White Musk (no label) is larger than the concentration specified by the Cosmetics Directive (0.001% for a leave-on sample).	[148]
9	A total of 20 commercial-scented plush toys (preserved in sealing packages before analysis).	Headspace (HS)-GC-MS	A total of 58 allergens were identified (natural extracts, which were unsuitable for a chromatography-based method, were not detected).	[149]
10	A total of 10 perfume products (7 eau de toilette, 2 aftershaves, and 1 eau de cologne) from the Swedish market.	2D HPLC-ESI-MS/MS	Detection of hydroxiperoxides of limone and linalool (limonene-2-hydroperoxide (Lim-2-OOH), linalool-6-hydroperoxide (Lin-6-OOH), and linalool7-hydroperoxide (Lin-7-OOH, with strong sensitization potency), the highest concentrations of the measured hydroperoxides (445 ± 23 ppm of total linalool hydroperoxides) being observed in one after-shave product, which is likely able to elicit skin reactions in already sensitized individuals.	[208]
11	A total of 7 different matrices: 5 were homemade (DWL, fabric softener, liquid laundry detergent, milky hair shampoo, day cream), 1 (powder detergent) was provided by a detergent manufacturer, and 1 was a natural raw material (Peru balsam, Nelixia, Antigua, Guatemala).	GC-MS	The standard addition protocol allowed the analysis of suspected allergens in the investigated matrices and allowed the quantification of all compounds (15 allergens) except farnesol and Lyral, within a concentration range of 50–100 mg/L.	[147]
12	A total of 62 commercialized perfumes.	GC×GC-qMS	A total of 56 (69 analytes including isomers) suspected chemically defined fragrance allergens in perfumes were investigated (the majority of the analytes could be determined under or above the 10 mg/kg regulated limit (88–100%).	[170]
13	Citrus oils.	LC-MS	Quantification of 15 furocoumarins.	[209]
14	Various cosmetic and personal care products.	UPC2-MS/MS	The analysis of the 24 regulated and 6 additional compounds (4 nonregulated cosmetic allergens and 2 potential carcinogenic compounds, methyl eugenol and 4-allyl anisole) was achieved using the Xevo TQD in MRM mode with APCI ionization (+/−), coupled to an ACQUITY UPC^2^ System, in a 7 min run. The method is more than six times faster than existing HPLC and GC methods, with 95% less solvent usage than existing HPLC methods.	[201]
15	A total of 7 oils issued from plants.	GC-MS	The chromatographic procedure seemed to be slightly longer; however, the conditions showed good resolution for about 200 terpenoid compounds determined in general essential oil studies. From the 25 standard allergens studied, 19 showed a retained DL (detection limit) < 13 mg/L, and 5 were = 30–50 mg/L. These variations are well explained by the form of the peaks. GC-MS is considered a good technique for the determination of volatile substances. Results were obtained with good repeatability.	[151]
16	A total of 4 commercial perfumes purchased in a local store (Messina, Italy).	GC-MS	The GC-MS method described is a rapid (<5 min) and effective screening tool in the determination of 26 allergens contained in mediumly complex perfumes. The twin-filtered MS library search procedure was shown to be a powerful tool for reliable compound identification. As for all monodimensional methods, it may fail when the number of sample volatiles greatly exceeds the peak capacity of a single column: the reliable qualitative/quantitative determination of 10–20 skin sensitizers amongst 500–1000 other volatiles would be an arduous task. A good result may be attained for a 200-component fragrance and a bad one for a 150-compound perfume. The most appropriate approach to be used, if a multiple-choice exists, strictly depends on the analyst’s experience and judgment.	[28]
17	Randomly chosen 18 cosmetic products—5 shampoos, 7 creams and lotions, 2 eau de toilette, 1 deodorant spray, 1 lipstick, 1 face powder and 1 soap bar.	GC-MS	The GC-MS method has been developed for the routine analysis of 11 fragrance substances in cosmetics: cinnamic alcohol, cinnamic aldehyde, eugenol, hydroxy citronellal, a-amyl cinnamic aldehyde, geraniol, isoeugenol, coumarin, dihydrocoumarin, citronellal, and citral. DL of all of the target fragrance substances were ~1 ppm.	[168]
18	A total of 5 commercial perfumes (P1–P5).	All FM GC×GC–qMS	The FM GC×GC–qMS method is sufficiently sensitive for all the 54 allergens considered. Moreover, and if required, the HR untargeted analysis of perfume constituents can be performed. The FM model proposed is a low-cost and effective alternative to cryogenic modulation; both the hardware and operational costs are somewhat limited, with many of the well-known benefits of GC×GC maintained.	[107]
19	Fragrance concentrates provided in blind by IFRA-member companies.	GC-MS GC×GC-MS	To determine a more realistic LOQ (limit of quantitation) in the context of a fragrance concentrate, a fragrance concentrate (FT) was spiked with all allergens at various levels between 10 and 500 mg/L and analyzed by a single laboratory. The accuracy profile shows that the mean bias remains less than 20% at all spiking levels down to 10 mg/L. For 90% of determinations, the expected bias should be less than 35% down to a level of 20 mg/L and between −49 and 77% at 10 mg/L (i.e., between 5 and 18 mg/L). This range remains acceptable to set the LOQ at 10 mg/L, in view of the suspected allergens analysis complexity. Fragrance concentrates are very complex mixtures; the occurrence of coelutions is frequent—the two-columns × three-ion option best minimizes the consequences of coelution on the determination of suspected allergens.	[188]
20	A total of 70 commercial perfumes and colognes.	GC-MS	Contents of 52 cosmetic ingredients belonging to 4 different types of ingredients: 6 preservatives, 12 synthetic musks, 26 fragrance allergens, and 8 phthalates can be determined in a single run. All samples contained some of the target ingredients. Several samples do not comply with the regulations concerning the presence of phthalates. Musk’s data confirmed the trend of the replacement of nitromusks by polycyclic musks, as well as the noticeable introduction of macrocyclic musks in the perfume’s composition. The prohibited musk moskene has been detected in one sample in an appreciable concentration. The average number of fragrance allergens is 12 per sample; values > 1% have been found in some samples. Preservatives data show that parabens, although ubiquitous in other cosmetic products, are not widely used in perfumery. In contrast, the presence of BHT is indeed widespread. Only about 38% of the perfumes were adequately labeled for the allergens tested.	[153]
21	A model mixture of volatiles and essential oils of different complexity (mint, lavender, and vetiver essential oils).	GC×GC MS GC×GC FID	Profiling and fingerprinting of medium- to highly complex samples of interest in the flavor and fragrance field was investigated. Capillary flow technology reverse-inject differential flow modulator was implemented with different column configurations (lengths, diameters, and stationary phase coupling) and detector combinations (MS and FID) to evaluate its potential in the quantitative profiling and fingerprinting of medium- to highly complex essential oils, and a parallel dual-secondary column dual-detection configuration that has shown to improve the information potential also with thermally modulated GC×GC platforms (MS for identification FID for quantitation) was tested. Experimental results demonstrate that careful tuning of column dimensions and system configurations yields improved (a) selectivity, (b) operable carrier gas linear velocities at close-to-optimal values, (c) 2D separation power by extending the modulation period, and (d) handling of overloaded peaks without dramatic losses in resolution and quantitative accuracy.	[173]
22	Different cosmetics (leave-on and rinse-off products) from national and international brands were purchased from local sources.	GC-MS	Accuracy, precision, linearity, and LODs were evaluated to assess the performance of the proposed method. Quantitative recoveries (>75%) were obtained, and RSD values were <10% in all cases. The quantification limits were well below those set by the international cosmetic regulations, making this multicomponent analytical method suitable for routine control. A total of 25 fragrance allergens were identified All the samples contained several of the target cosmetic ingredients, with an average number of seven. The total fragrance allergen content was, in general, relatively high, even in baby care products, with values close to or up to 1%, for several samples, although the actual European Cosmetic Regulation was fulfilled.	[176]
23	A total of 60 household commodities, including perfumes, lotions, hair care products, and household cleaners, were purchased from retail stores in Albany, New York.	GC-MS	Concentrations of HHCB, AHTN, and HHCB-lactone in consumer products ranged from <5 ng/g to over 4000 μg/g, <5 ng/g to 451 μg/g, and <5 ng/g to 217 μg/g, respectively. The highest concentrations were found in perfumes, body creams, lotions, and deodorants. The results suggest that a wide variety of source materials exist for HHCB and AHTN and that these materials are used on a daily basis.	[177]
24	Different cosmetic products.	GC-MS	The analysis of suspected volatile allergens in products containing high-molecular-weight or nonvolatile compounds such as plant extracts, solid and liquid detergents, and shampoos was performed. Based on PTV injection with ALEX and GC-MS, nonvolatile matrices are retained in the liners filled with PDMS foam, while good analytical performance for the target solutes is preserved. This approach drastically shortens and simplifies the sample preparation step. The method also gives quasi-quantitative analyte recoveries for all solutes with the exception of methyl-2-octynoate and methyl-2-nonynoate. For various nonvolatile matrices, a single external calibration can be used, while for the two mentioned esters, internal standardization is presently carried out. Analyzing all target compounds in the different matrices with one single method is impossible; therefore, we proposed at various meetings to classify the different matrices into four classes. Class I consists of samples that contain volatile or semivolatile solutes, typically eluting on an apolar column between n-decane (retention index 1000) and n-docosane (retention index 2200). Class II also consists of samples containing only volatile and semivolatile solutes, but their complexity is very high (A100 solutes) and/or the concentration range is very broad (e.g., very low concentration of a target compound next to a very high concentration of matrix compound). Class III comprises nonvolatile samples (solutes eluting after n-hexacosane). Class IV matrices are finished products like soaps, liquid and solid detergents, etc. In these samples, the solutes are typically present at relatively low concentrations, while the matrix can be quite complex due to the presence of glycols and surfactants.	[156]
25	A total of 10 samples (several moisturizing and antiwrinkle creams and lotions, hand creams, and sunscreen and after-sun creams).	GC-MS	A new method based on solid-phase dispersion-pressurized liquid extraction (PLE) followed by GC-MS has been developed for the determination of 26 suspected fragrance allergens (all the regulated in the EU Cosmetics Directive amenable by GC, as well as pinene and methyl-eugenol) in cosmetic samples. The study revealed the presence of suspected allergens in all the analyzed samples, and half of the samples contained an elevated number of them.	[157]
26	A total of 26 cosmetic products (creams, emulsions, lotions, gels for the skin, bath and shower preparations, deodorants, hair-setting, hair-cleansing, and hair-conditioning products, shaving products, and sunbathing products).	GC-MS	MSPD and GC-MS were used for the rapid determination of 18 plasticizers (phthalates and adipates), 7 polycyclic musks, and 5 nitromusks, which makes a total of 30 targets in both rinse-off and leave-on cosmetic formulations. A total of 25 out of 30 targets were detected in the samples. The most frequently found compounds were galaxolide and tonalide, reaching concentrations above 0.1% (1000 g·g^−1^) and diethyl phthalate (between 0.7 and 357 g·g^−1^). The presence of banned substances such as dibutyl phthalate, diisobutyl phthalate, dimethoxyethyl phthalate, benzylbutyl phthalate, diethylhexyl phthalate, diisopentyl phthalate and dipentyl phthalate, musk ambrette, and musk tibetene was confirmed in 16 of the 26 personal care products (62%).	[154]
27	A broad range of cosmetics and personal care products (shampoos, body milk, moisturizing milk, toothpaste, hand creams, gloss lipstick, sunblock, deodorants, and liquid soaps, among others).	GC-MS	A practical, simple, and low-cost sample GC-MS and GC-MS/MS method has been developed for the rapid simultaneous determination of 38 cosmetic ingredients, 25 fragrance allergens, and 13 preservatives. The final miniaturized process required the use of only 0.1 g of sample and 1 mL of organic solvent for the final extract ready for analysis. The concentration levels ranged from the sub-parts per million to the parts per million. Several fragrances (linalool, farnesol, hexylcinnamal, and benzyl benzoate) have been detected at levels >0.1% (1000 g·g^−1^). With regard to preservatives, phenoxyethanol was the most frequently found additive, reaching a relatively high concentration (>1500 g·g^−1^) in 5 cosmetic products. BHT was detected in 8 samples, in 2 of them (a baby care product and a lipstick) at high concentrations (>1000 g·g^−1^). In 3 leave-on samples, methyl paraben was also found at high levels (>1700 g·g^−1^). Finally, triclosan was found at the maximum concentration limit (0.3%) laid down by the European regulation in 2 deodorant samples, and the total paraben concentration was close to the maximum concentration permitted (0.8%) in one leave-on sample (body milk).	[98]
28	Personal care products and sanitation products (*n* = 82) were obtained through the cooperation of several volunteers. The samples were divided into six categories: sanitation products (*n* = 14), perfumes (*n* = 19), deodorants (*n* = 4), hair care products (*n* = 12), shower and bath products (*n* = 18), and body lotions (*n* = 15).	GC-MS	An overview of the synthetic musk levels in 6 different personal care product categories was performed. Especially body lotions, perfumes, and deodorants contain high levels of synthetic musks. Maximum concentrations of HHCB, AHTN, MX, and MK were 22 mg·g^−1^, 8 mg·g^−1^, 26 μg·g^−1^, and 0.5 μg·g^−1^, respectively. By combining these results with the average usage of consumer products, low-, medium-, and high-exposure profiles through dermal application could be estimated. HHCB was the highest contributor to the total amount of synthetic musks in every exposure profile (18–23,700 lg·d1). Exposure to MK and MX did not increase substantially (10–20-fold) between low- and high-exposure profiles, indicating that these compounds cover a less broad range. In comparison, exposure to HHCB and AHTN increased up to 10,000 fold between low and high exposure.	[159]
29	A total of 73 household commodities were purchased in Kumamoto, Japan: perfumes (n¼13), fabric softeners (n¼11), shampoos (n¼11), body lotions (n¼9), body soap (n¼5), antiperspirants (n¼5), laundry detergents (n¼4) toilet deodorants (n¼4), body fragrances (n¼2), hair liquid (n¼2), sunscreen (n¼2), dish cleaner (n¼2), tooth powder (n¼2), and bath cleaner (n¼1).	GC-MS	Occurrence and concentrations of macrocyclic-, polycyclic-, and nitro musks in cosmetics and household commodities collected from Japan. The high concentrations and detection frequencies of Musk T, habanolide, and exaltolides were found in commercial products, suggesting their large production and usage in Japan. Polycyclic musks, HHCB and OTNE, also showed high concentrations in cosmetics and products. The estimated dairy intakes of Musk T and HHCB by the dermal exposure to commercial products were 7.8 and 7.9 μg/kg/day in humans, respectively, and perfume and body lotion are dominant exposure sources. The dairy intakes of HHCB by dust ingestions were 0.22 ng/kg/day in humans, which were approximately 5 orders of magnitude lower than those of dermal absorption from commercial household commodities.	[160]
30	Fragrance-free cosmetic samples (creams, body lotions, oils) were bought from commercial shops in Basel and stored at room temperature until preparation for recovery experiments (adding allergens in the range of 10 mg/kg). Additionally, for quality control, a hand cream (oil in water emulsion) of known fragrance content in the range of 4–15 mg/kg was used as a reference sample.	GC-MS	SEC combined with GC-MS was developed for the quantitation of 24 restricted allergenic fragrance compounds in cosmetic samples. Fragrance calibration has to be performed with propyl acetate as a solvent containing a constant proportion of matrix components. With the exception of hydroxycitronellal (66 ± 5%), all compounds showed good recovery rates in the range of 90–120%. The mean accuracy (relative error) was 1 ± 10% for all 24 compounds in five spiked creams (10 mg/kg per allergen) and 8 ± 34% in a reference sample (4–15 mg/kg). The most significant benefit compared to other methods is the flexible clean-up with SEC, which allows the determination of an extensive range of compounds in difficult matrices with GC-MS.	[180]
31	Personal care products were purchased from retail stores in Porto, Portugal: body and hair washes (*n* = 5), toilet soaps (*n* = 1), skin moisturizers (*n* = 4), roll-on deodorants (*n* = 1), and toothpaste (*n* = 1).	GC-MS	The developed and validated method using QuEChERS extraction followed by GC-MS was applied to the analysis of 12 samples, which revealed musk concentrations ranging from 2 ng/g (toothpaste) to 882,340 ng/g (perfumed body lotion).	[186]
32	Cosmetic samples from national and international brands were purchased from local markets in Beijing.	GC-MS	A total of 7 synthetic musks (musk ambrette, musk tibetene, musk moskene, musk ketone, musk xylene, phantolide, and tonalide) are extracted and prepurified by a mixture solution of water and isopropanol from cream and separated and purified by tandem columns containing SLE column and LC-Alumina-N SPE column. This pretreatment method combined with GC-MS/MS technology has been proved to be precise, accurate, and applicable to the routine analysis of 7 synthetic musks residues in cream samples.	[189]
33	A total of 12 commercially available essential oils were purchased from local chemical material stores in Taiwan. A total of 5 culinary herbs (holy basil, sweet basil, thyme, laurel, and rosemary) and 5 spices (cumin, cinnamon, nutmeg, cardamon, and clove) were purchased from local food retail stores in Kaohsiung, Taiwan. Samples of 4 commercially available aromatherapy massage oil products were obtained from randomly selected cosmeceutical stores in Taiwan.	GC-MS	A simple and quick sample preparation method was developed and used for preconcentration and extraction of six phenylpropenes, including anethole, estragole, eugenol, methyl eugenol, safrole, and myristicin, from oil samples by dual dispersive liquid–liquid microextraction. GC-MS was used for the determination and separation of compounds. Several experimental parameters affecting extraction efficiency were evaluated and optimized. For all analytes (10–1000 ng/mL), the limits of detection (S/N 3) ranged from 1.0 to 3.0 ng/mL; the limits of quantification (S/N 10) ranged from 2.5 to 10.0 ng/mL; and enrichment factors ranged from 3.2 to 37.1 times. Within-run and between-run relative standard deviations (*n* = 6) were less than 2.61% and 4.33%, respectively. Linearity was excellent, with determination coefficients (r2) above 0.9977. The experiments showed that the proposed method is simple, effective, and environmentally friendly for analyzing phenylpropenes in oil samples.	[163]
34	A total of 3 perfumes, 2 anti-hair loss products, 1 post-depilation mousse, 1 cream deodorant, and 3 different cream samples (body, sun, and hand creams).	GC-qMS	Qualitative and quantitative analysis of 24 volatile compounds listed as suspected allergens in cosmetics by the European Union was performed. The applicability of a headspace (HS) autosampler in combination with GC equipped with a programmable temperature vaporizer (PTV) and a qMS detector is explored. The method showed good precision and accuracy, and it is rapid, simple, and highly suitable for the determination of suspected allergens in different cosmetic products.	[181]
35	Commercial perfume samples and a cosmetic product (body cream) were obtained from a supermarket.	GC/GC×GC-MS	Suspected fragrance allergens were determined in cosmetic products using a combination of whole evaporation-dynamic headspace (FEDHS) with selectable GC/GC×GC-MS using capillary flow technology (CFT) and low thermal mass GC (LTM-GC). The FEDHS approach allows the nondiscriminating extraction and injection of both apolar and polar fragrance compounds without contamination of the analytical system by high-molecular-weight nonvolatile matrix compounds. The system is highly flexible and easy to use, and was applied to all classes of cosmetic samples, including water-containing matrices such as shower gels or body creams.	[182]
36	The samples, analyzed for their content on illegal skin bleaching agents, were taken by inspectors affiliated with the Belgian federal public service “Animal, Plant and Food Directorate-General” (DG4) and the Belgium Federal Agency for Medicinal and Health Products (FAMHP) and were also used for this study.	GC-MS	A new headspace GC-MS method was capable of analyzing 24 volatile allergenic fragrances in complex cosmetic formulations, such as hydrophilic and lipophilic creams, lotions, and gels. This method was successfully validated using the total error approach. The trueness and precision deviations for all components were smaller than 8%, and the expectation tolerance limits did not exceed the acceptance limits of ±20% at the labeling limit—used to analyze 18 cosmetic samples that were already identified as being illegal on the EU market for containing forbidden skin-whitening substances.	[165]
37	Different personal care products: a hand cream water-in-oil (*w*/*o*), an eau de perfume, a shower gel, and an orange oil, and they were purchased from a local drugstore.	LC-MS	There is an advantage of the Direct EI LC-MS interface for the quantitation of principal components, as well as for the identification of unknown/undeclared ingredients Commercially available products were diluted with methanol and injected directly into a nano-LC column. Limonene, linalool, and citral were selected as target compounds because of their use as fragrances in toiletry and detergent products. Selected compounds are not detected with ESI because of their poor or very low response. No matrix effects were observed, and the repeatability was excellent even after several weeks of operation. The product’s composition was investigated in full scan mode to determine the presence of unknown or nonlisted ingredients.	[195]
38	A total of 10 randomly selected perfumes and similar products.	LC-MS	An LC-MS method for quantitative analysis of the potential oak moss allergens atranol and chloroatranol in perfumes and similar products was developed and validated. LOD for atranol and chloroatranol were 5.0 ng/mL and 2.4 ng/mL, respectively; the method based on LC-MS and LC-MS/MS with ESI in negative mode and SRM allowed the identification of these compounds at concentrations below those causing allergic skin reactions in oak-moss-sensitive patients. The recovery of chloratranol from spiked perfumes was 96 ± 4%. Low recoveries (49 ± 5%) were observed for atranol in spiked perfumes, indicating ion suppression caused by matrix components.	[197]
